# Distinct colitis-associated macrophages drive NOD2-dependent bacterial sensing and gut homeostasis

**DOI:** 10.1172/JCI190851

**Published:** 2025-10-02

**Authors:** Gajanan D. Katkar, Mahitha Shree Anandachar, Stella-Rita C. Ibeawuchi, Ella G. McLaren, Megan L. Estanol, Kennith Carpio-Perkins, Shu-Ting Hsu, Celia R. Espinoza, Jane E. Coates, Yashaswat S. Malhotra, Madhubanti Mullick, Vanessa Castillo, Daniella Vo, Saptarshi Sinha, Pradipta Ghosh

**Affiliations:** 1 Department of Cellular and Molecular Medicine,; 2 Biomedical Sciences Graduate Program, School of Medicine,; 3 Department of Pathology,; 4 HUMANOID Center of Research Excellence,; 5 Department of Pediatrics, and; 6 Department of Medicine, UCSD, La Jolla, California, USA.

**Keywords:** Gastroenterology, Immunology, Microbiology, G proteins, Innate immunity, Macrophages

## Abstract

Single-cell studies have revealed that intestinal macrophages maintain gut homeostasis through the balanced actions of reactive (inflammatory) and tolerant (noninflammatory) subpopulations. How such balance is impaired in inflammatory bowel diseases (IBDs), including Crohn’s disease (CD) and ulcerative colitis (UC), remains unresolved. Here, we define colon-specific macrophage states and reveal the critical role of noninflammatory colon-associated macrophages (niColAMs) in IBD recovery. Through trans-scale analyses—integrating computational transcriptomics, proteomics, and in vivo interventional studies—we identified GIV (*CCDC88A*) as a key regulator of niColAMs. GIV emerged as the top-ranked gene in niColAMs that physically and functionally interacts with NOD2, an innate immune sensor implicated in CD and UC. Myeloid-specific GIV depletion exacerbates infectious colitis, prolongs disease, and abolishes the protective effects of the NOD2 ligand muramyl dipeptide in colitis and sepsis models. Mechanistically, GIV’s C-terminus binds the terminal leucine-rich repeat 10 (LRR 10) of NOD2 and is required for NOD2 to dampen inflammation and clear microbes. The CD-associated *1007fs* NOD2 variant, which lacks LRR 10, cannot bind GIV, which provides critical insights into how this clinically relevant variant impairs microbial sensing and clearance. These findings illuminate a critical GIV•NOD2 axis essential for gut homeostasis and highlight its disruption as a driver of dysbiosis and inflammation in IBD.

## Abstract 2

<article data-scroll-anchor=”false” data-testid=”conversation-turn-3” data-turn=”user” dir=”auto” tabindex=”-1”> This study identifies a critical interaction between GIV and NOD2 in gut macrophages, revealing their importance in maintaining gut health and preventing inflammatory bowel diseases. </article>

## Introduction

Intestinal macrophages are critical for gut development, immunity, and repair (1). Single-cell studies have revealed that gut homeostasis relies on the dynamic interplay between 2 antagonistic macrophage subpopulations: inflammatory “accelerators” and noninflammatory “brakes” (2, 3). An imbalance in these subpopulations can lead to uncontrolled gut inflammation, as observed in inflammatory bowel diseases (IBDs) such as Crohn’s disease (CD) and ulcerative colitis (UC) (4, 5). However, precisely defining these subpopulations and understanding their roles in health and disease, and the molecular mechanisms that control the same, remain significant challenges (6).

**Figure 1:**
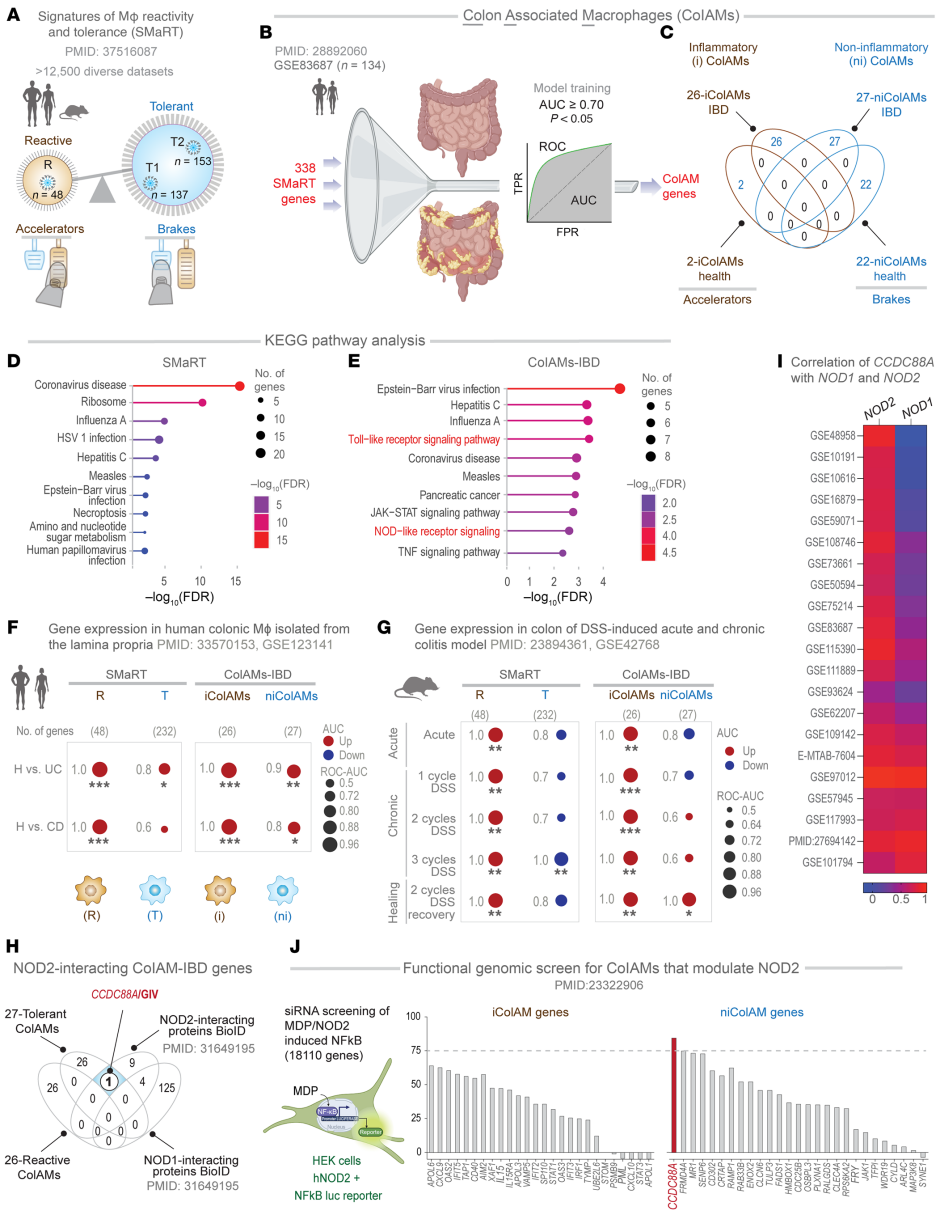
Identification of CCDC88A as a putative NOD2 modulator in IBD-associated macrophages. (**A**) Key steps of a previously published workflow (3) used to develop the computational model of macrophage continuum states—SMaRT, which identifies invariant gene clusters representing reactive (R) and tolerant (T1, T2) states across >12,500 diverse transcriptomic datasets. The schematic illustrates their opposing roles: reactive macrophages act as accelerators, while tolerant states serve as brakes, working antagonistically to fine-tune inflammatory responses to perceived threats. (**B**) Key steps used to refine SMaRT in the context of the gut mucosa and derive ColAM signatures using a dataset (24) comprising both healthy and IBD samples. SMaRT genes were trained to derive a subset of ColAMs that could classify healthy versus IBD samples, achieving an AUC ≥ 0.7 and *P* ≤ 0.05 (Fisher’s exact test). (**C**) iColAM- and niColAM-defining genes identified in healthy and IBD samples. (**D** and **E**) Kyoto Encyclopedia of Genes and Genomes pathway enrichment analysis for SMaRT (**D**) and the ColAM-IBD (**E**) gene sets. (**F** and **G**) The receiver operating characteristic–AUC (circle size) and regulation (red, up; blue, down) for classifying healthy versus CD and healthy versus UC in human (H) colonic lamina propria (GEO GSE123141) (**F**) and DSS-induced acute, chronic, and healing phases of murine colitis models (**G**). Classification was based on macrophage gene signatures of reactivity (R) and tolerance (T), identified in the SMaRT model and the iColAMs and niColAMs, used independently. *P ≤ 0.05; **P ≤ 0.01; ***P ≤ 0.001, Welch’s 2-sample unpaired *t* test. (**H**) Venn diagram of ColAM genes identified in **B** and **C** with NOD1 and NOD2 interactors identified by independent studies. CCDC88A (GIV; white circle) emerges as a NOD2-specific interactor linked to tolerant ColAMs. (**I**) Correlation coefficient of normalized gene expression of CCDC88A with NOD2 and NOD1 across independent transcriptomic datasets of healthy and IBD tissues. (**J**) MDP/NOD2-induced NF-κB activity observed during a functional genomic (siRNA-based) screen. The impact of depletion of iColAM and niColAM genes is presented. The dashed line marks 75% enhancement relative to MDP-stimulated controls. Luc, luciferase; Mϕ, macrophage.

Recent advances in artificial intelligence and machine learning–guided transcriptomics have addressed this challenge by enabling the analysis of diverse macrophage states across both bulk and single-cell datasets (3, 7–10). Among these approaches, Boolean implication networks have emerged as a robust method with a decade-long track record (7, 8, 11, 12) of identifying universally conserved gene expression patterns (or “invariants”). These patterns remain consistent despite the variability introduced by tissue heterogeneity, circadian rhythms, metabolic states, species diversity, perturbations, stimuli, and disease conditions (7, 8, 11, 12). Using this network approach on a dataset of pooled isolated monocytes and macrophages representing the greatest possible diversity, we recently defined Signature of Macrophage Reactivity and Tolerance (SMaRT) (Figure 1A and Supplemental Data Set 1; supplemental material available online with this article; https://doi.org/10.1172/JCI190851DS1) as a conserved 338-gene signature representing macrophage continuum states, across the physiologic and pathologic spectra of reactivity and tolerance (3). We showed that while the conventional M1/M2 classification fails to capture the diversity, plasticity, and continuum of macrophage states in tissue during homeostasis and disease, the SMaRT model–derived definitions remain robust and consistently outperform other emerging classification schemes across contexts (3).

Here we sought to refine the SMaRT model in the context of IBD. We hypothesized that these definitions would yield robust classification and functional insights into the colitic environment. First, we formally define 2 macrophage subpopulations in the colon—inflammatory colon-associated macrophages (iColAMs) and noninflammatory colon-associated macrophages (niColAMs) —both in health and IBD. We find that tolerant niColAMs are essential for dampening inflammation and resolving infections, making them critical for recovery from IBD. We subsequently identify a previously unappreciated yet consequential physical and functional coupling in IBD-associated niColAMs between the innate immune sensor nucleotide-binding oligomerization domain containing protein 2 (NOD2) and Gα-interacting vesicle-associated protein (GIV), also known as Girdin. NOD2, also known as NLRC2, belongs to the nucleotide-binding domain and leucine-rich repeat family and functions as an intracellular pattern recognition receptor (PRR) for muramyl dipeptide (MDP) derived from pathogens. NOD2 coordinates bacterial clearance and confers immunity (4), all while mounting a controlled inflammatory program that involves the dampening of NF-κB (13) activity that is TLR2/4 dependent (14–18). GIV, on the other hand, is a multimodular signal transducer and the prototypical member of the nonreceptor guanine nucleotide exchange modulator (GEM) (19) family of proteins. Unlike the canonical GPCR/G protein pathway, in which G proteins engage exclusively with ligand-activated GPCRs, GEMs like GIV bind and modulate G protein activity downstream of myriad cell-surface receptors (20–22). Of relevance here, GIV is a ubiquitously expressed molecule that is highly expressed in immune cells such as macrophages and serves as a brake for the cell-surface PRR TLR4 and modulates macrophage inflammatory responses to LPS (22) and gut barrier integrity during aging (23), cancer (23), and in IBD (7). Its gene, *CCDC88A*, has emerged as a key determinant of macrophage polarization in the SMaRT model (3). We demonstrate that GIV interacts dynamically with NOD2 to facilitate microbial sensing and clearance while also suppressing inflammation. This protective mechanism is disrupted in the most clinically significant IBD-associated NOD2 risk variant, highlighting its relevance to disease pathology. These insights shed new light on the molecular pathways underlying gut homeostasis and the progression of IBD, offering potential therapeutic avenues for restoring balance in macrophage subpopulations.

## Results

### Identification of distinct subpopulations of colon-associated macrophages.

To contextualize the SMaRT model (Figure 1A and Supplemental Data Set 1) within the human gut, and specifically in IBD, we refined it using the largest, high-quality, full-thickness colon tissue transcriptomic dataset available for IBD (accession GSE83687) (24)—the only dataset of its kind. Because the original model was built using purified macrophages and monocytes from diverse tissues, we assumed that refinement using bulk RNA-Seq data would preserve a subset of macrophage-specific genes from the SMaRT model that are most relevant to IBD. Briefly, we used a machine learning–based classifier on 338 SMaRT signature genes (3) (Supplemental Data Set 1) to identify the classification accuracy of each of the SMaRT signature genes on healthy versus IBD-affected colon tissues (Figure 1B). This allowed us to formally define colon-associated macrophages (ColAMs) in health as those expressing a core set of 24 genes (*n* = 2 expressed highly in reactive iColAMs; *n* = 22 expressed highly in tolerant niColAMs) (Figure 1C), and in IBD, as those expressing a distinct set of 53 genes (*n* = 26 expressed highly in reactive iColAMs; *n* = 27 expressed highly in tolerant niColAMs) (Figure 1C). It is noteworthy that the brakes and accelerators in health are distinct from IBD (Figure 1C, and see Supplemental Data Set 2). The ColAM genes had AUC values greater than 0.70 (Figure 1B and Supplemental Data Set 2) in discriminating between healthy and IBD-affected colon tissues (including UC and CD) (Supplemental Data Set 2). Kyoto Encyclopedia of Genes and Genomes pathway enrichment analysis revealed that model refinement led to enrichment of colitis-relevant pathways, including Toll-like receptor, NOD2, and TNF signaling (compare Figure 1, D and E; and see Supplemental Data Set 2 for gene lists).

When we tested their ability to distinguish healthy from colitis samples, the 53-gene ColAM signature (used independently as 26-gene iColAMs and 27-gene niColAMs) performed consistently better than the original SMaRT model (3) in both human (vs. UC/CD) (Figure 1F) and murine (vs. dextran sodium sulfate [DSS], a chemical colitogen) (Figure 1G) datasets. Leveraging a high-quality murine dataset of DSS-induced acute and chronic colitis (25), we found that iColAMs and niColAMs may be induced in temporally distinct patterns. iColAMs were induced acutely and persisted throughout the various DSS models, whereas niColAMs were induced exclusively in a chronic model in which injury was repetitive in the form of 2 cycles of DSS followed by 3 weeks of recovery/washout, which is believed to better recapitulate the relapsing-remitting nature of IBD (Figure 1G).

### NOD2 may functionally couple with CCDC88A in niColAMs.

*NOD2*, located on chromosome 16, remains the most replicated genetic association in IBD, with a mean allelic odds ratio of 3.1 across studies (26, 27) and a well-established, though mechanistically debated, role in IBD pathogenesis (4, 28–32). Persistent controversy surrounds how NOD2 functions and how its variants drive colitis in both UC and CD (32–36). Given the enrichment of NOD signaling in IBD-associated ColAMs (Figure 1E), we investigated NOD-centric cellular processes in iColAMs and niColAMs.

Overlaying iColAM and niColAM gene clusters with a published NOD1/2 interactome, as determined by BioID Proximity-Dependent Biotin Identification (37), we identified a single candidate interactor: GIV, encoded by *CCDC88A* (Figure 1H). Notably, *CCDC88A* is part of the niColAM gene signature, which emerges during the recovery phase of DSS-induced colitis (Figure 1G). Its expression correlates with *NOD2*—but not *NOD1*—across 21 independent cohorts (Figure 1I) and is elevated in intestinal macrophages from patients with UC and CD compared with healthy control individuals (Supplemental Figure 1A).

We next leveraged a genome-wide siRNA screen in HEK293T cells (38) that assessed MDP-induced hyperactivation of NF-κB. While NOD2 variants are known to impair bacterial clearance and disrupt NF-κB activation (13, 29, 38, 39), paradoxically, the gut mucosa of patients with IBD often shows heightened NF-κB activity (40–44). Loss-of-function NOD2 variants, such as the CD-associated *1007fs* (45), are also known to impact the severity of the disease course in UC (46). Based on these observations, NOD2 is believed to restrict activation of the NF-κB pathway by TLR2/4 (14–18), and its dysfunction causes runaway inflammation, thereby increasing the risk of colitis. Consistent with these observations, the functional-genomic screen revealed that among all iColAM and niColAM-genes, depletion of *CCDC88A* within the niColAM cluster (genes presumed to be critical for reducing inflammation) emerged as the most consequential perturbation that increases NF-κB activity (Figure 1J).

These findings suggest that CCDC88A may functionally couple with NOD2 to restrain inflammation in ColAMs, providing a strong rationale to investigate the protective niColAM state during colitis recovery.

### GIV is required for MDP/NOD2-mediated bacterial clearance and controlled inflammation.

**Figure 2:**
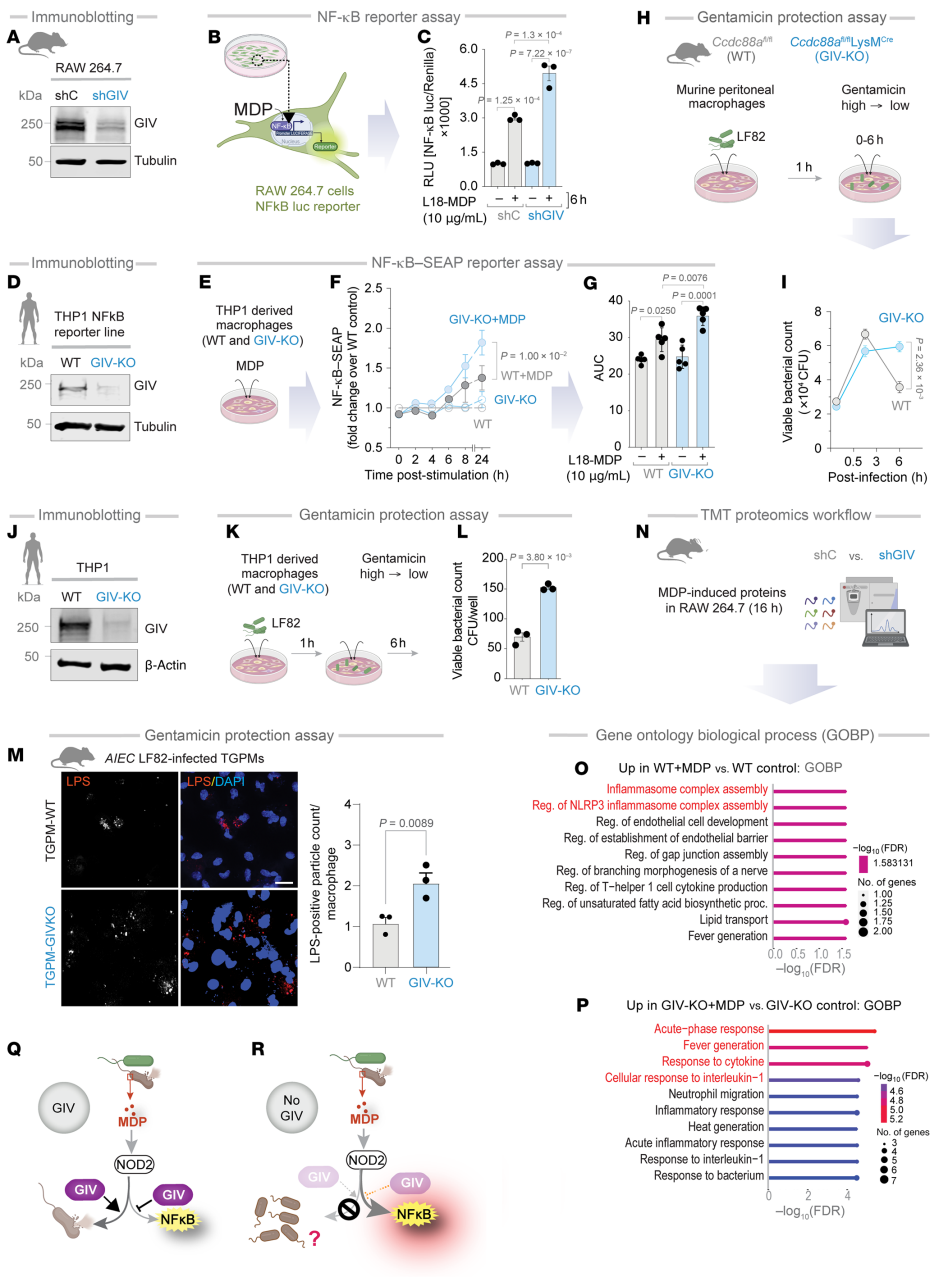
GIV dampens inflammation and promotes bacterial clearance in MDP-stimulated macrophages. (**A**) Immunoblot of control (shC) or shGIV RAW 264.7 cells. (**B** and **C**) Workflow of the NF-κB reporter assay in RAW 264.7 cells (**B**). Bar graphs display the fold change in NF-κB activity (**C**). (**D**) Immunoblot of WT (control) and GIV-KO THP1-NF-kB SEAP reporter cell line–derived macrophages. (**E**–**G**) Workflow of the NF-κB reporter assay in CRISPR-depleted human THP-1 cells expressing an NF-κB activity–tracking reporter (**E**). Line graphs (**F**) and AUC bar graphs (**G**) display the fold change in NF-κB activity relative to WT control. (**H**) Workflow of the bacterial clearance assay (**H**). (**I**) Line graphs show the viable bacterial counts in the peritoneal macrophages. (**J**) Immunoblot of WT or GIV-KO THP1 monocyte–derived macrophage. (**K**) Workflow of the gentamicin protection assay in THP1 cells. (**L**) Bar graphs show the viable bacterial counts in the THP1 monocyte–derived macrophage. (**M**) Immunofluorescence images display representative fields of TGPMs challenged with live AIEC-LF82 (MOI 1:30) for 1 hour. Scale bar: 20 μM. Bar graphs display quantification of intracellular AIEC-LF82; *n* = 4–6. (**N**) Workflow for multiplexed proteomics analyses. (**O** and **P**) Bar graphs showing biological process as determined by Gene Ontology biological process (GOBP) analysis (red indicates the pathways cited in the text). (**Q** and **R**) Schematics summarizing findings in cells with GIV (**Q**) and without GIV (**R**). All results are displayed as mean ± SEM (*n* = 3 biological replicates). Significance was tested using 1-way ANOVA with Tukey’s test (**C** and **G**), 2-way ANOVA with Tukey’s test (**F** and **I**), and 2-tailed Student’s *t* test (**L** and **N**). *P* ≤ 0.05 is considered significant. Luc, luciferase; Reg, regulation.

Given its recently identified role in modulating macrophage responses (22), we asked if GIV may be a functional modulator of the cytosolic sensor NOD2. To study the role of GIV in MDP/NOD2-induced inflammatory responses in macrophage in vitro, we used 4 cell-based models. (a) GIV-depleted (shGIV) RAW 264.7 murine macrophage cells; this previously validated cell model displays approximately 85%–90% depletion of GIV protein by immunoblotting (22) (Figure 2A). (b) THP1 NF-κB–secreted embryonic alkaline phosphatase (SEAP) reporter human macrophage lines depleted or not of approximately 90% GIV protein (by CRISPR; Figure 2D). (c) Thioglycolate-induced murine peritoneal macrophages (TGPMs) isolated from myeloid-specific conditional GIV KO mice, generated previously (22) by crossing GIV floxed mice to *LysM*cre mice and confirmed to have approximately 85%–90% depletion of GIV protein. And (d) THP1 human macrophage lines depleted or not of approximately 90% GIV protein (by CRISPR) (Figure 2J).

In GIV-depleted RAW macrophages, MDP/NOD2-induced NF-κB activity was significantly elevated (Figure 2, B and C), as determined by luciferase reporter assays. Dynamic NF-κB reporter assays in THP1 reporter cells further confirmed the findings, adding robustness to the results (Figure 2, E–G). These findings were corroborated in HeLa cells (Supplemental Figure 1, B–D), a cell line commonly used to study NOD2-dependent processes in plasmid transfection settings (47, 48). Briefly, compared with control cells, GIV-depleted HeLa cells (by CRISPR) (Supplemental Figure 1B) had significantly higher MDP/NOD2-induced NF-κB activity, confirming the role of GIV in dampening NF-κB activity. Consistent with its role in dampening inflammation, GIV depletion in TGPMs led to approximately a 3- to 4-fold increase in proinflammatory cytokines (IL-1β, IL-6, and TNF-α), as measured by ELISA (Supplemental Figure 1, E and F). Hyperinduction of proinflammatory cytokines was accompanied by a concomitant suppression of the anti-inflammatory cytokine IL-10 (Supplemental Figure 1, E and F). These cytokine profiles were consistent with gene expression patterns assessed via qPCR (Supplemental Figure 1G).

When TGPMs were infected with adherent-invasive *E*. *coli* strain-LF82 (*AIEC*-LF82), isolated from patients with CD (49), GIV-KO TGPMs exhibited delayed bacterial clearance compared with WT controls (Figure 2, H and I). Similarly, GIV-KO THP1 cells reproduced these findings (Figure 2, K and L). Immunofluorescence imaging further confirmed that GIV-KO TGPMs retained significantly higher numbers of pathogenic *AIEC*-LF82 bacteria (Figure 2M).

Tandem mass tag–based quantitative proteomics of GIV-depleted RAW macrophages revealed distinct proteomic differences after 16 hours of MDP stimulation (Figure 2N). While control cells activated robust NOD2-dependent signaling and inflammasome assembly (Figure 2O), GIV-depleted cells had an acute-phase response and heightened expression of proinflammatory cytokines (Figure 2P).

Together, these results identify GIV as a critical mediator of MDP/NOD2 signaling. GIV is essential for maintaining a balanced pro- and anti-inflammatory cytokine response and promoting effective bacterial clearance. In its absence, macrophages exhibit exaggerated NF-κB–driven inflammation but fail to clear bacteria efficiently (Figure 2, Q and R), suggesting that GIV’s role in microbial clearance may be independent of its modulation of NF-κB signaling.

### GIV is required for phagolysosomal fusion.

To understand why GIV-deficient macrophages retain higher intracellular bacterial loads despite heightened NF-κB activity (Figure 2R), we next investigated whether GIV plays a direct, NF-κB–independent role in bacterial clearance. Because NOD2-dependent response to degraded bacteria requires the phagosomal membrane potential and the activity of lysosomal proteases (50), we hypothesized that GIV may facilitate phagolysosomal (PL) fusion.

**Figure 3:**
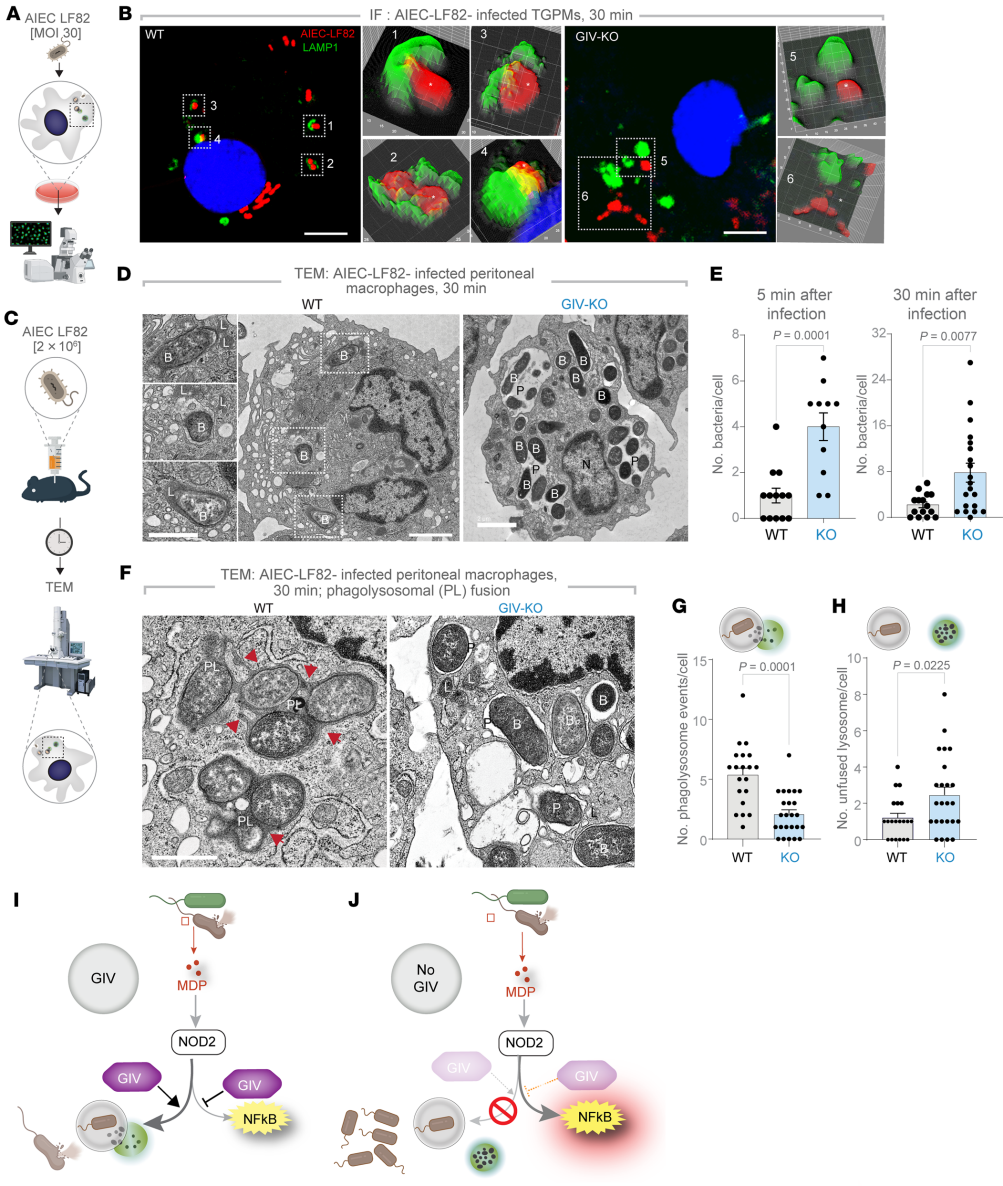
GIV is required for PL fusion and bacterial clearance. (**A** and **B**) Workflow for immunofluorescence studies of AIEC-LF82–challenged TGPMs (**A**) and representative images (**B**) showing the proximity of AIEC-LF82 (red) to LAMP1-positive lysosomes (green). Insets show magnified, 3D-rendered versions of boxed regions, created using ImageJ. Scale bars = 5 μm. (**C**) Workflow for TEM studies of infected peritoneal macrophages in **D**–**H**. (**D**) Representative TEM images showing bacterial abundance. Scale bar: 2 μm. (**E**) Bar graphs quantifying the number of bacteria per cell at 5 and 30 minutes after infection. (**F**) High-magnification TEM images highlighting PL fusion events (arrowheads). (**G** and **H**) Bar graphs display the number of events per cell (**G**) and number of unfused lysosomes per cell (**H**); *n* = 2 repeats. B, bacteria AIEC-LF82N; L, lysosome; nucleus; P, phagosome; PL, phagolysosome. (**I** and **J**) Summary of findings in cells with (**I**) or without (**J**) GIV. All TEM quantifications were based on ~20–30 fields; *n* = 2 independent biological repeats. Results are presented as mean ± SEM. Significance was determined using 2-tailed Student’s *t* test; *P* ≤ 0.05 is considered significant.

We used 2 complementary approaches to test this. First, we challenged TGPMs in vitro with *AIEC*-LF82 and assessed the spatial proximity of internalized bacteria to LAMP1-positive lysosomes by confocal immunofluorescence microscopy (Figure 3A). In control (WT) cells, *AIEC*-LF82 bacteria were frequently found near LAMP1-positive structures (Figure 3B), suggesting efficient delivery of phagosomes to lysosomes. By contrast, GIV-deficient macrophages showed a marked reduction in bacteria-lysosome proximity (Figure 3B), suggesting disrupted lysosomal targeting.

Second, we used quantitative transmission electron microscopy (TEM) to visualize PL fusion events and quantify bacterial burden over time (Figure 3C). GIV-deficient macrophages harbored visibly more intracellular *AIEC*-LF82 compared with WT controls (Figure 3D), both at 5- and 30-minutes after infection (Figure 3E), confirming impaired bacterial clearance. TEM imaging also revealed stark ultrastructural differences: while WT cells exhibited numerous PL fusion events (Figure 3F, arrowheads), GIV-deficient macrophages showed markedly fewer fusion events (Figure 3G) and retained more unfused lysosomes (Figure 3H), suggesting a defect in phagosome maturation and lysosome engagement, but not lysosome biogenesis.

These findings define a mechanistically distinct, NF-κB–independent role for GIV in promoting PL fusion. In its absence, bacterial clearance fails despite heightened inflammatory signaling, underscoring GIV’s dual function: restraining inflammation via NF-κB modulation and promoting pathogen elimination through lysosomal trafficking (Figure 3, I and J).

### GIV-KO mice develop dysbiosis and exacerbated and protracted Citrobacter-induced colitis.

**Figure 4:**
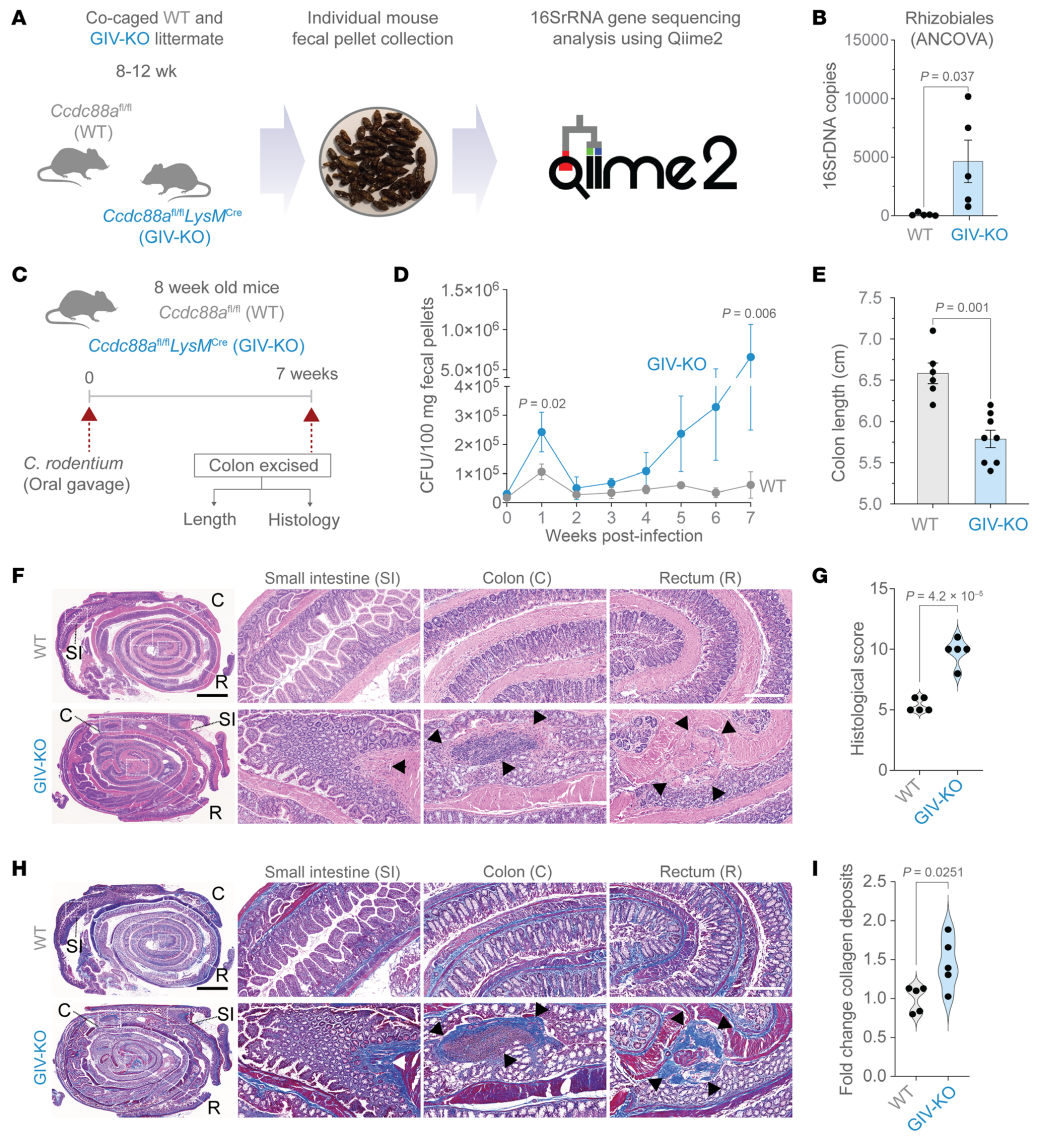
A mouse model of dysbiosis, impaired microbial clearance, patchy chronic transmural ileocolitis, and fibrosis. (**A** and **B**) Schematic (**A**) and bar graph (**B**) display the process and outcome of a 16S fecal microbiome analysis (using the QIIME 2 multi-omics data science framework) at baseline in 10-week-old, myeloid-specific (LysMCre) GIV-KO mice and their control littermates (WT). *n* = 5 mice in each group. (**C**–**I**) Panel describe the experimental design (**C**) and findings (**D**–**I**) in an infectious colitis model of GIV-KO and control littermates induced using *Citrobacter rodentium* (initially named *C*. *freundii* biotype 4280 (88); strain name DBS100); 5 × 10^8^ CFU/200 μL/mouse. GIV-KO, *n* = 8; WT, *n* = 6. Findings are representative of 2 independent repeats. (**D**) Line graphs display the bacterial burden in fecal pellets over 7 weeks after the initial oral gavage. (**E**) Bar graph displays the differences in colon length. H&E-stained (**F**) or trichrome-stained (**H**) images representative of Swiss rolls of the entire intestinal tract are shown. Scale bar: 2.5 mm. Magnified fields of the rectum (R), colon (C), and small intestine (SI) of the corresponding boxed regions are shown. Scale bar: 250 μm. Arrows show regions of transmural inflammation or crypt distortion; immune infiltrates (**F**) correspond also to transmural fibrosis (**H**). Segments in between these patches appear normal. Bar graphs show the histology index (89) (**G**) based on submucosal inflammation, percent area involved, inflammatory infiltrates in LP and crypt hyperplasia, and the degree of fibrosis (**I**), as assessed by H&E and trichrome staining of 5 WT and 5 GIV-KO mice. All results are displayed as mean ± SEM. Significance was tested using 2-tailed Student’s *t* test. Only significant *P* values (≤ 0.05) are shown.

To investigate the role of GIV in vivo, we used a myeloid-specific GIV-KO (*Ccdc88a*^fl/fl^/*LysM*^Cre^) model (see Methods) (22). We found that these mice spontaneously develop dysbiosis by approximately 8–12 weeks (Figure 4, A and B, and Supplemental Figure 2). Notably, the strain Rhizobiales—uniquely associated with patients with CD and absent in healthy control individuals (*P* = 0.037) (51)—was detected in 100% of GIV-KO mice (*n* = 5 of 5) but was undetectable in control littermates (Figure 4, A and B, and Supplemental Figure 2).

Upon *Citrobacter* challenge (Figure 4C), GIV-KO mice exhibited an increased acute fecal bacterial load (Figure 4D; first week) and an abnormal delay in bacterial clearance, leading to chronic infection (Figure 4D; seventh week). These mice also demonstrated hallmark features of chronic colitis, including colon shortening (Figure 4E); patchy transmural inflammation affecting the small intestine, colon, and rectum (Figure 4, F and G); as well as focal muscle hypertrophy and collagen deposition (Figure 4, H and I). Because the absolute numbers of macrophages, and specifically, M2 macrophages—defined by established conventional markers CD68 and CD163, respectively (52–54)—were comparable between *Citrobacter*-infected control and GIV-KO intestinal tissues (Supplemental Figure 3, A–D), we conclude that GIV deficiency impairs the healing functions of ColAMs without affecting macrophage trafficking or polarity-defining M2 markers at the site of infection.

Collectively, these findings highlight a critical role of GIV in bacterial clearance and the resolution of inflammation. Its absence promotes dysbiosis and chronic infectious colitis, underscoring GIV’s essential role in maintaining intestinal immune homeostasis.

### Protective MDP/NOD2 signaling is abolished in myeloid-specific GIV-KO mice.

**Figure 5:**
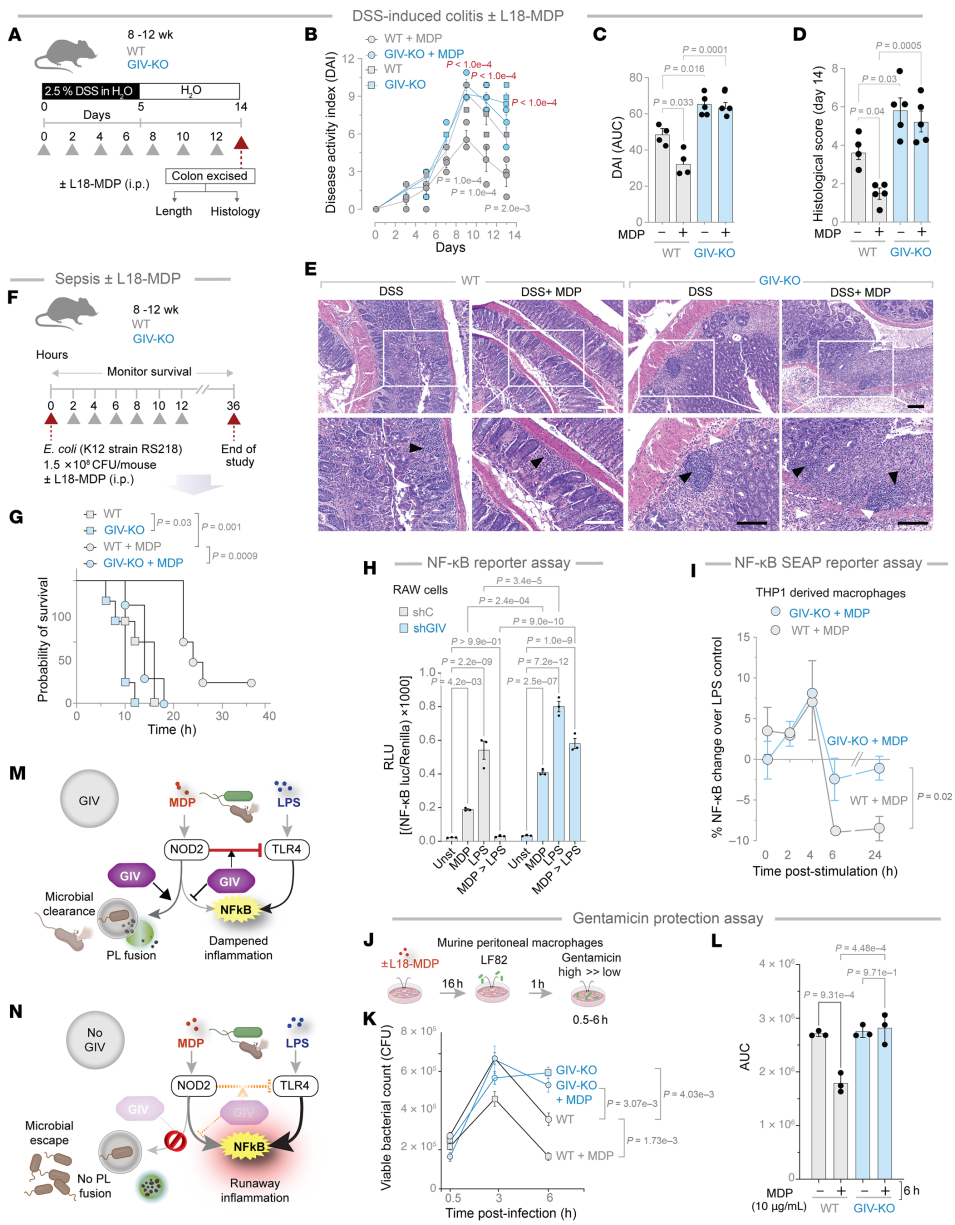
GIV-KO mice and cells are insensitive to the protective actions of MDP/NOD2 signals. (**A**) Schematic displays the study design for DSS-induced colitis. GIV-KO, *n* = 5; WT, *n* = 5. Findings are representative of 2 independent repeats. Gray arrowheads denote the alternate-day administration of MDP (100 μg/mouse/d). (**B**) Disease activity index (DAI), calculated for the days 3, 5, 7, 9, 11, and 13 after DSS administration, which accounts for stool consistency (0–4), rectal bleeding (0–4), and weight loss (0–4). *P* values in gray and red text represent the statistical significance of WT versus WT plus MDP and WT plus MDP versus GIV-KO plus MDP, respectively. (**C**) Data in **B** as AUC. (**D**) Histological score on day 14, as assessed by a well-accepted methodology (90) of analyzing H&E-stained distal colons from the mice. (**E**) Representative images are displayed. Arrowheads point to regions of crypt destruction and/or inflammatory infiltrates. Scale bar: 200 μm. (**F**) Schematic displays the sepsis study design in which 8 mice in each group were treated with *E*. *coli* and MDP simultaneously, followed by periodic checks for death (arrowheads). (**G**) Kaplan-Meier plot displays the percentage of the cohort that survived at those time points. GIV-KO, *n* = 8; WT, *n* = 8. Findings are representative of 2 independent repeats. (**H**) Impact of MDP (10 μg/mL) priming on 100 ng/mL LPS-induced NF-κB activity. (**I**) Percentage change in 100 ng/mL LPS-induced NF-κB activity in WT and GIV-KO cells primed with MDP (10 μg/mL). (**J**) Schematic displays the experimental setup for bacterial clearance. (**K**) Viable bacterial counts in the macrophages. (**L**) Data in **K** as the AUC. (**M** and **N**) Schematic summarizing findings in cells with (**M**) or without (**N**) GIV. All results are displayed as mean ± SEM. Significance was tested using 1-way ANOVA with Tukey’s test (**C**, **D**, **H**, and **L**), 2-way ANOVA with Tukey’s test (**B**, **I**, and **K**), and Mantel-Cox log-rank test (**G**). *P* ≤ 0.05 is considered significant. Luc, luciferase; Unst, unstimulated.

Prior studies have shown that pretreatment with MDP ameliorates infection or bacteremia (55, 56), fatality in sepsis (57), and chemical-induced colitis (e.g., with trinitrobenzene sulfonic acid, 2,4,6-trinitrobenzenesulfonic acid [TNBS], DSS) (15). We asked if these protective actions of MDP require GIV. Compared with WT controls, we found that the GIV-KO mice developed significantly worse DSS-induced acute colitis (Figure 5A), as determined by disease activity index (Figure 5, B and C) and histological composite scores accounting for deformation of colon crypts and increased immune infiltration in the colon (Figure 5, D and E). The latter is a composite score of stool consistency, weight loss, and the presence of fecal blood (22, 58, 59). Pretreatment with MDP ameliorated the severity of colitis in WT mice but not GIV-KO mice (Figure 5, B–E). Because the absolute numbers of CD68^+^ M1 and CD163^+^ M2 macrophages were comparable between control and GIV-KO DSS-exposed intestinal tissues (Supplemental Figure 3, E–H), GIV deficiency appears to impact MDP-induced ColAM properties without affecting macrophage trafficking or polarity-defining M2 markers at the site of inflammation.

Similar results were observed in the case of *E*. *coli*–induced sepsis (Figure 5F); the fatality rate was higher in GIV-KO mice than in WT controls (Figure 5G). Pretreatment with MDP reduced deaths in WT, but not GIV-KO, mice (Figure 5G). These findings demonstrate that GIV is required for the protective MDP/NOD2 signaling in the setting of infection or inflammation.

Prior studies have shown that MDP priming of NOD2 protects cells from excessive inflammation induced by LPS (15, 60). To determine if this protective effect requires GIV, we used shGIV RAW 264.7 murine macrophages and WT controls to assess NF-κB activation after LPS stimulation, with or without MDP pretreatment. In WT cells, MDP pretreatment significantly reduced NF-κB activation, but this protective effect was markedly compromised in GIV-depleted cells (Figure 5H). The findings were also reproduced in CRISPR-depleted human THP-1 cells expressing an NF-κB activity–tracking reporter, enabling continuous monitoring of signaling dynamics. The presence of GIV was required for sustained suppression of NF-κB activity, evident as early as 6 hours and maintained through 24 hours (Figure 5I). Similar findings were observed in HeLa cells, which express the MD2 coreceptor essential for LPS/TLR4 signaling (48, 61–65), albeit at low levels (66). In control HeLa cells, MDP pretreatment reduced NF-κB activation significantly, both with endogenous NOD2 (Supplemental Figure 1H) and exogenously overexpressed NOD2 (Supplemental Figure 1I). In cells without GIV, this protective effect was either diminished (Supplemental Figure 1H) or virtually abolished (Supplemental Figure 1I). Additionally, when MDP-primed TGPMs were infected with the pathogenic *AIEC*-LF82 strain (49), MDP treatment accelerated bacterial clearance in WT cells but not in KO TGPMs (Figure 5, J–L).

These findings demonstrate that GIV is essential for protective MDP/NOD2 signaling, which counteracts LPS/TLR4-driven proinflammatory NF-κB signaling (Figure 5M). Without GIV, NF-κB signaling becomes excessive, and bacterial clearance is delayed and impaired (Figure 5N).

### MDP/NOD2 signals induce niColAMs and GIV is required for such induction.

**Figure 6:**
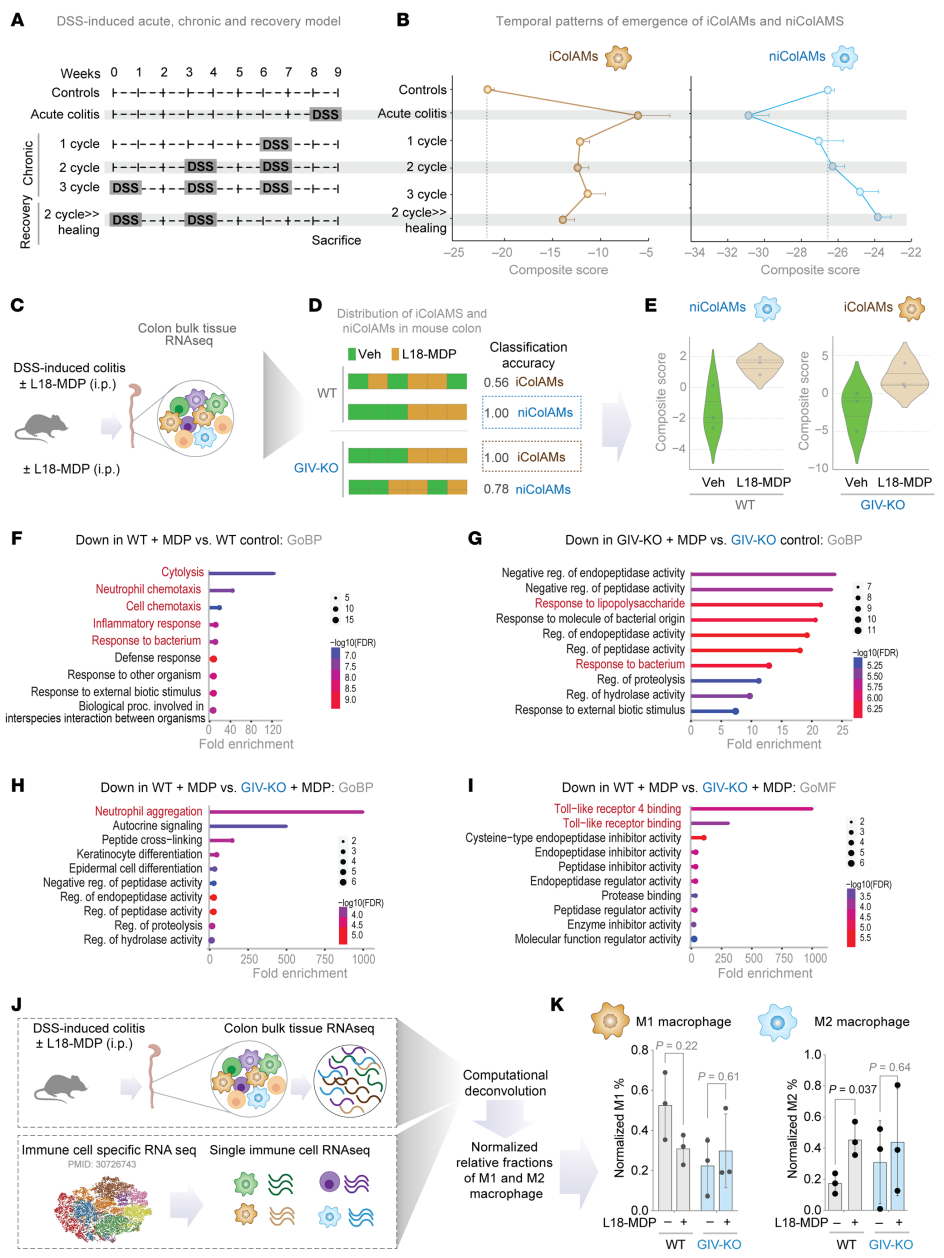
GIV is required for the emergence of healing niColAMs in MDP-treated WT mice. (**A**) Study design of DSS-induced acute, chronic, and recovery phases in mouse models of colitis (C57/BL6; all WT). (**B**) Line graphs display the temporal patterns of the emergence of iColAMs and niColAMs in the colon samples in **A**. The gray dotted line indicates the composite scores of iColAM and niColAM genes in the control mice. (**C**) Study design for DSS-induced colitis in WT versus GIV mice (*n* = 3 each). See also Figure 5, A–E, for the detailed study design and disease pathology. (**D**) Bar plots show the classification accuracy of composite scores derived from iColAM and niColAM gene signatures in DSS-challenged mouse samples, comparing with or without L18-MDP treatment groups. Classification strength within each cohort is measured using receiver operating characteristic AUC analyses. (**E**) Violin plots show composite scores for niColAMs (for blue border in **D**) in WT and iColAMs (for brown border in **D**) in GIV-KO mice, treated with or without L18-MDP. (**F** and **G**) Gene Ontology Biological Process (GOBP) pathway enrichment analyses of genes downregulated in WT (**F**) or GIV-KO (**G**) mice treated with L18-MDP compared to their respective untreated controls. (**H** and **I**) GOBP (**H**) and Go Molecular Function (GO MF; **I**) analyses of genes downregulated in L18-MDP-treated WT vs GIV-KO samples. (**J** and **K**) Schematic (**J**) of bulk RNA sequencing in silico deconvolution analysis of distal colons from DSS-treated mice in **C**. Bar plots (**K**) show normalized percentage abundances of M1 and M2 macrophages in WT and GIV-KO mice, with and without MDP treatment. Statistics: *P*-values were calculated using an unpaired multiple *t*-test (**K**). *P*-value ≤ 0.05 is considered as significant. GOBP, Gene Ontology biological process; proc, processes; Reg, regulation; veh, vehicle.

To assess the role of MDP/NOD2 signaling in modulating iColAM and niColAM populations, we analyzed publicly available transcriptomic datasets of DSS-induced colitis spanning the acute, chronic, and recovery phases (Figure 6A), using composite gene signatures of i iColAM and niColAM subsets. iColAM populations were elevated during the acute phase but declined during the chronic and recovery phases (Figure 6B), while niColAMs showed the opposite trend: their numbers increased abundance during recovery (Figure 6B).

Because GIV is required for the protective effects of MDP/NOD2 signaling in DSS-induced colitis (Figure 5, A–E), we next asked whether this protection arises from MDP’s ability to promote early induction of niCoIAMs, thereby accelerating recovery from acute colitis. We analyzed colon transcriptomes from WT and GIV-KO mice treated with DSS-induced colitis, with or without MDP treatment (Figure 6C). In WT mice, a composite niColAM score robustly distinguished MDP-treated WT colon tissues from untreated WT controls (classification accuracy = 1) (Figure 6, D and E). This indicates early upregulation of healing niColAMs by MDP—earlier than anticipated from phase-specific trends (Figure 6B)—and potentially accelerating recovery. By contrast, in GIV-KO mice, MDP treatment elevated iColAM scores (also with classification accuracy = 1) (Figure 6, D and E), consistent with the observed exacerbation of inflammatory responses (Figure 5, A–E). Gene Ontology analysis of differentially expressed genes corroborated these findings, revealing activation of protective and reparative programs in MDP-treated WT mice but not in GIV-KO mice (Figure 6, F–I, and see Supplemental Data Set 3 for full genes list).

To determine whether the healing niColAM population aligns with conventional noninflammatory macrophage (M2) populations, we performed bulk RNA-Seq deconvolution. This revealed a close transcriptional resemblance between niColAMs and M2-like (anti-inflammatory) macrophages, which were enriched in MDP-treated WT, but not GIV-KO, mice (Figure 6, J and K).

Together, these results underscore the essential role of GIV in enabling MDP/NOD2-mediated protection in vivo, by selectively promoting the emergence of healing niColAMs that counterbalance pro-inflammatory iColAMs and restore tissue homeostasis.

### The GIV•NOD2 interaction is direct and dynamically regulated by MDP.

**Figure 7:**
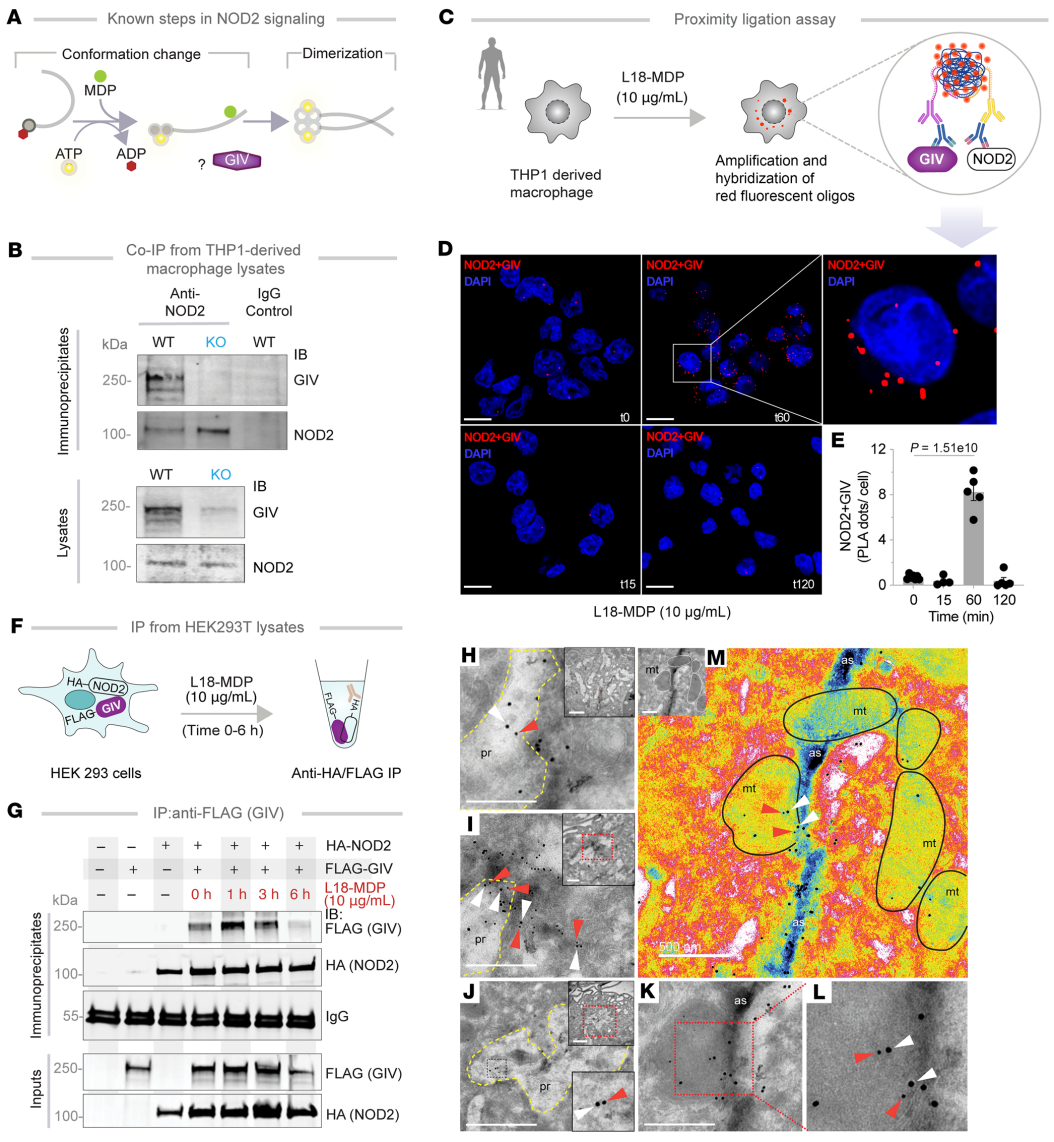
NOD2 and GIV colocalize and interact in cells. (**A**) Schematic displays key steps in MDP-induced NOD2 signaling. In resting cells, ADP-bound inactive NOD2 exists in an autoinhibited conformation. In resting cells, ADP-bound NOD2 is autoinhibited. Upon ligand (MDP) stimulation, ADP is exchanged for ATP, stabilizing ligand binding, inducing conformational change and “opening” of the LRR module, followed by NOD2 dimerization and assembly of signaling complexes. (**B**) IP of full-length endogenous NOD2 from THP1-derived macrophage lysates. Immune complexes were analyzed for bound GIV by IB; input lysates were probed for NOD2 and GIV. (**C**) Study design of PLA. (**D**) Representative confocal images show colocalization of GIV and NOD2 in THP1-derived macrophages challenged with MDP for 0–120 minutes. Scale bar: 10 μm. (**E**) Quantification from ~20–30 randomly imaged fields; *n* = 4–5 repeats. *P* value determined by 1-way ANOVA, followed by Tukey’s test for multiple comparisons and indicated with *P* shown above bars. *P* ≤ 0.05 is considered as significant. (**F**) Schematic depicts study design of IP from lysates of HEK293T cells. HA-tagged NOD2 was IP with anti-HA mAb from equal aliquots of lysates of HEK293T cells coexpressing GIV-FLAG and HA-NOD2, stimulated (+) or not (–) with MDP for indicated time points. (**G**) IP complexes and input lysates were analyzed for NOD2 and GIV by IB. (**H**–**M**) TEM micrographs display representative images of colocalization of GIV (white arrowheads; 18 nm gold particles) and NOD2 (red arrowheads; 12 nm gold particles) on TGPMs challenged with live AIEC-LF82 (MOI 1:30) for 1 hour. NOD2 colocalization within particle-rich cytoplasmic structures (pr), with membrane-associated GIV on actin strands (as; pseudo-colored blue) and swollen mitochondria (mt; outlined in black) with degraded cristae in **M**. Scale bar: 500 nm; 1 μm (**H** and **J**, insert). Oligo, oligonucleotide.

NOD2 typically exists in an inactive, ADP-bound conformation stabilized by intramolecular interactions (67, 68). Upon binding its ligand, MDP, NOD2 undergoes conformational changes that facilitate ADP-to-ATP exchange, self-oligomerization, and downstream signaling (67, 68) (Figure 7A).

To determine whether GIV physically interacts with NOD2, we performed co-IP experiments and found that full-length, endogenous GIV and NOD2 form complexes in THP1-derived macrophages (Figure 7B). In situ proximity ligation assay using Abs against the native proteins confirmed this interaction and revealed that the abundance of GIV•NOD2 complexes is enhanced by MDP stimulation, peaking around 1 hour after treatment (Figure 7, C–E). Co-IP assays using exogenously expressed, epitope-tagged GIV and NOD2 proteins further validated this interaction and its ligand-dependent dynamic regulation. Whether GIV-FLAG or HA-NOD2 was used as bait, assembled GIV•NOD2 complexes were detected in immune complexes within ~1–3 hours after MDP stimulation, and declined by approximately 6 hours (Figure 7, F and G, and Supplemental Figure 4A), underscoring the temporally regulated nature of the interaction.

To visualize the ultrastructural context of the GIV•NOD2 complex assembly, we performed immunogold electron microscopy on TGPMs 1 hour after MDP stimulation. Using 18 nm and 12 nm gold-conjugated Abs against GIV and NOD2, respectively, we observed NOD2 colocalizing with membrane-associated GIV, predominantly along actin filaments (Figure 7, H–L) within particle-rich cytoplasmic structures (69), which contain polyubiquitinated proteins and proteasomes (Figure 7, H and J), and around swollen, morphologically abnormal mitochondria (Figure 7M).

Together, these findings demonstrate that the GIV•NOD2 interaction is both direct and dynamically regulated by MDP. The complex associates with the membrane and cytoskeletal elements, supporting its potential role in NOD2-mediated signaling and cellular responses (70).

### The C-terminus of GIV directly binds the LRR domain of NOD2.

**Figure 8:**
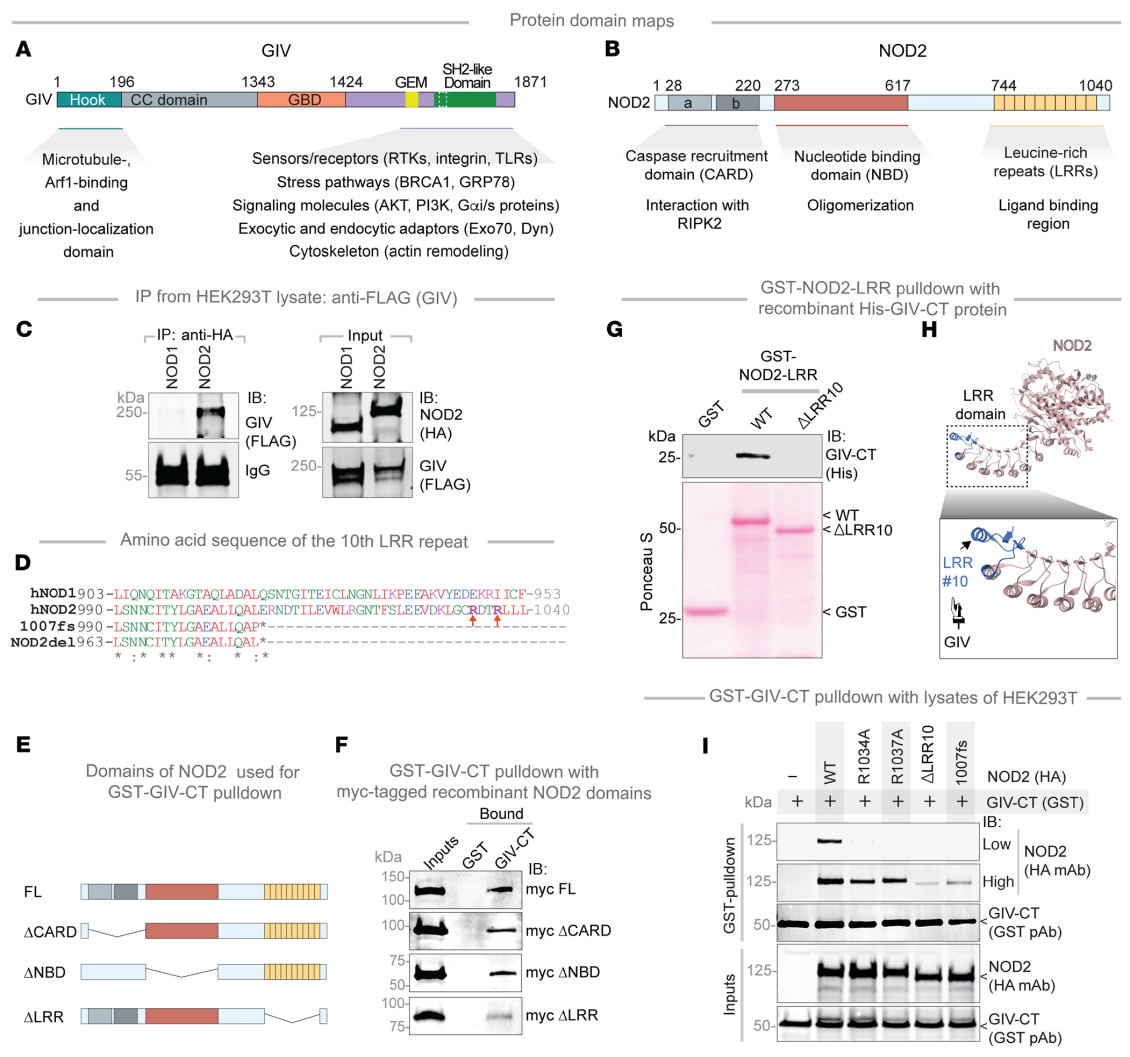
The NOD2(LRR)•GIV(C-term) interaction is direct and ligand-dependent. (**A** and **B**) Schematic depicts the domain maps of GIV (**A**) and NOD2 (**B**) and highlights established functions and interactions facilitated by the domains. (**C**) HA-tagged NOD1/2 proteins were IP from equal aliquots of lysates of HEK293T cells cotransfected with GIV and either NOD1 or NOD2 using anti-HA mAb. IP complexes and input lysates were analyzed for NOD1/2 and GIV by IB. (**D**) An alignment of the aa sequence of the 10th LRR repeat of human (h) hNOD1, hNOD2, the CD-risk associated NOD2 variant (NOD2-*1007fs*), and the deletion (del) mutant generated in this work (NOD2-del) is shown. Residues mutated in this study to evaluate potential participating residues in the NOD2•GIV interaction are highlighted. (**E**) Schematics indicate the domains of NOD2 that were used to generate myc-tagged recombinant proteins for use in GST-pull-down assays. (**F**) Equal aliquots of recombinant myc-NOD2 domains (~3 μg; inputs) were used in pulldown assays with immobilized GST and GST-GIV. Myc-tagged NOD2 was visualized by IB using anti-myc Ab. (**G**) GST-pulldown assay was carried out using GST NOD2-LRR proteins as indicated, and bound His-GIV-CT is assessed. (**H**) Schematic highlights the terminal LRR repeat (blue) of NOD2 which binds GIV. (**I**) GST–GIV-CT was pulled down using glutathione beads from equal aliquots of lysates of HEK293T lysates coexpressing GST–GIV-CT (aa 1660–1870; mammalian p-CEFL vector) and either WT or HA-NOD2 mutants predicted to disrupt NOD•GIV binding. IP complexes and input lysates were analyzed for NOD2 and GIV-CT by IB, using anti-HA (NOD2) and anti-GST (GIV-CT) Abs. RTK, receptor tyrosine kinase.

GIV is a large, multimodular scaffold protein (*n* = 1,870 aa) with several defined interaction domains (Figure 8A). NOD2, in contrast, contains 3 major domains: the caspase recruitment domain (CARD), nucleotide binding domain (NBD), and LRR (Figure 8B). While NOD1 and NOD2 share structural similarities, co-IP analyses revealed that GIV specifically binds to NOD2 but not NOD1 (Figure 8C), indicating that the GIV•NOD2 interaction is selective.

Given that the approximately 210 aa C-terminal (CT) module of GIV mediates interactions with a variety of receptors and sensors via short linear motifs (SLIMs) (Figure 8A), we tested whether this region was sufficient for NOD2 binding. Indeed, GIV-CT–bound NOD2 (Supplemental Figure 4B) but did not interact with NOD1 (Supplemental Figure 4B), further confirming specificity.

To identify the domain of NOD2 responsible for GIV binding, we performed co-IP assays using NOD2 deletion mutants lacking the CARD (ΔCARD), NBD (ΔNBD), or LRR (ΔLRR) domains (Figure 8, E and F). These studies showed that GIV binding was independent of the CARD and NBD domains and instead required the LRR domain (Figure 8, E and F). Notably, deletion of the terminal repeat in the LRR domain (ΔLRR10) virtually abolished GIV binding (Figure 8, G and H). These findings were corroborated by co-IP using full-length proteins (Supplemental Figure 4E) and glutathione *S*-transferase (GST) pull-down studies using GIV-CT (Figure 8I and Supplemental Figure 4F). Moreover, site-directed mutagenesis of key arginine residues in NOD2 (R1034 and R1037) (Figure 8D), which stabilize its terminal LRR (Figure 8H), reduced GIV binding (Figure 8I).

Together, these results demonstrate that the terminal LRR repeat of NOD2 is essential for its interaction with the CT region of GIV, providing insights into the molecular basis of their functional coupling (Figure 8, G–I).

### GIV fails to bind the CD-associated NOD2 1007fs variant, which lacks the terminal LRR.

**Figure 9:**
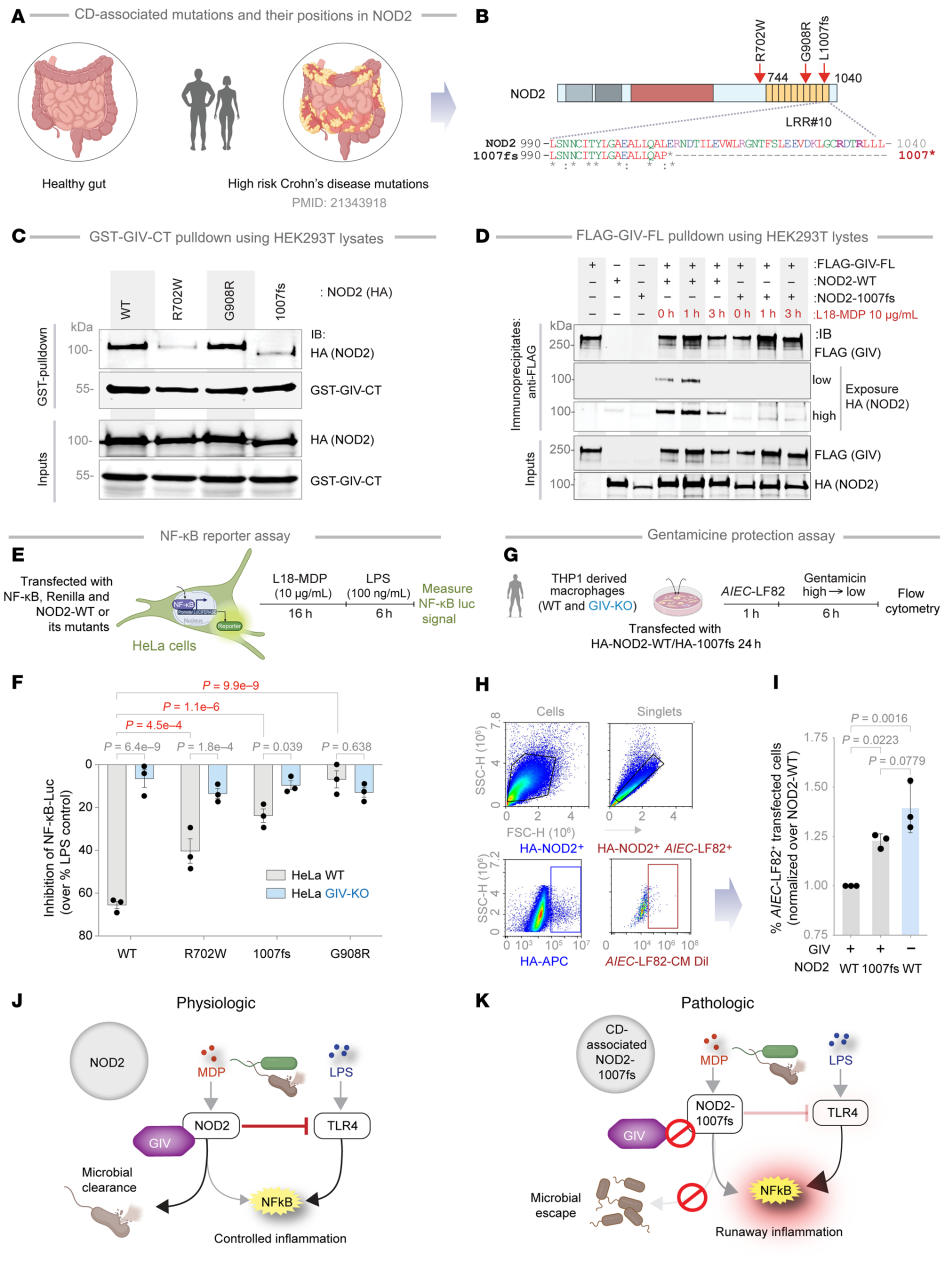
Characterization of the NOD2•GIV interface exploiting CD-associated NOD2 mutants. (**A** and **B**) Schematic shows CD-associated mutations (**A**) and their positions in NOD2, depicted as a domain map, and alignment (**B**) of the aa sequence of the 10th LRR repeat of human NOD2 and the CD-risk associated NOD2 variant (NOD2-*1007fs*). Residues mutated to evaluate potential participating residues in the NOD2•GIV interaction are highlighted. (**C**) GST–GIV-CT was pulled down using glutathione beads from equal aliquots of lysates of HEK293T coexpressing GST–GIV-CT (aa 1660–1870; mammalian p-CEFL vector) and WT or 3 indicated CD-risk associated variants of HA-NOD2. Bound NOD2 proteins and similar expression of GIV-CT was assessed by IB using anti-HA (NOD2) and anti-GST (GIV-CT) Abs. (**D**) FLAG-tagged GIV was IP with anti-FLAG mAb from equal aliquots of lysates of HEK293T cells expressing GIV-FLAG and either WT or *1007fs* variant of HA-NOD2, stimulated (+) or not (–) with MDP for the indicated time points. IP complexes and input lysates were analyzed for NOD2 and GIV by IB, using anti-HA (NOD2) and anti-FLAG (GIV-CT) Abs. (**E**) Workflow for assessing NF-κB activity. (**F**) NF-κB reporter assay in HeLa cells. Cells were preincubated with MDP (10 μg/mL) and then stimulated with LPS (100 ng/mL) and the percentage change of NF-κB activity was detected using a dual-cell reporter assay. (**G**) Workflow for assessing bacterial clearance via flow cytometry. (**H**) The flow cytometry panel detects CM-Dil–labeled AIEC-LF82 bacteria (MOI 1:30) in THP1-derived macrophages transfected with HA-NOD2-WT or *1007fs* mutant. (**I**) AIEC-LF82 bacterial load normalized to NOD2-WT. *P* ≤ 0.05 was considered significant, 1-way ANOVA followed by Tukey’s multiple comparison test. (**J** and **K**) Schematic summarizing key findings in this work. Magenta solid and interrupted lines indicate the GIV-dependent impact on NOD2. (**J**) In physiology, bacterial sensing and signaling by NOD2 requires GIV to limit inflammation. (**K**) In pathology, dysregulated inflammation results when either WT NOD2 cannot bind GIV (e.g., GIV is low or absent) or when the CD-risk associated *1007fs* variant cannot bind GIV. *P* values were calculated using 1-way ANOVA with Tukey’s test (**I**) and 2-way ANOVA with Tukey’s test (**F**) and indicated with *P* values are shown. *P* ≤ 0.05 was considered significant.

We next examined whether GIV binding is altered by CD-associated NOD2 variants (*R702W*, *G908R*, and *1007fs*), which collectively account for approximately 80% of mutations associated with CD susceptibility (Figure 9A) (32, 71). These mutations affect residues located within or near the LRR domain (Figure 9B). Co-IP assays revealed that 2 variants, R702W and 1007fs (Figure 9C), did not bind to the CT region of GIV. Notably, these variants are associated with high disease penetrance (~100%) (Supplemental Table 1). By contrast, the G908R variant, which disrupts the MDP-binding interface (72, 73), retained GIV binding comparable to NOD2-WT (Figure 9C). Further studies confirmed that the *1007fs* variant remained incapable of binding GIV even upon MDP stimulation (Figure 9D).

To assess the functional consequences of these binding defects, we examined how the CD-risk variants modulate NF-κB signaling in response to LPS after MDP priming. In luciferase reporter assays, MDP pretreatment significantly suppressed LPS-induced NF-κB activation in the presence of NOD2-WT (Figure 9, E and F), conferring 65% protection. However, this protective effect was reduced in cells expressing the NOD2 variants (Figure 9F, *P* values in red). For instance, while NOD2-WT conferred approximately 65% protection, the NOD2-*1007fs* variant (~20% protection) or other GIV-binding deficient mutants, *R702W* and *G908R* showed approximately 45% and approximately 7% protection, respectively (Figure 9F, *P* values in red). In GIV-KO cells, suppression by NOD2-WT dropped from approximately 65% to approximately 10%, further confirming the role of GIV in this protective response (Figure 9F, *P*-values in gray). The residual approximately 10%–20% suppression observed in conditions lacking GIV•NOD2 coupling (e.g., with NOD2-*1007fs* or in GIV-KO cells) suggests minor contributions from GIV-independent mechanisms or a consequence of endogenous NOD2.

To investigate the impact of GIV on NOD2-dependent microbial clearance, we performed gentamicin protection assays in THP1-derived macrophages transfected with either NOD2-WT or the *1007fs* variant, followed by infection with adherent-invasive *E*. *coli* (*AIEC*-LF82) (Figure 9, G and H). Macrophages expressing NOD2-WT efficiently cleared bacteria, whereas those with the *1007fs* variant showed impaired clearance, reflected by elevated intracellular bacterial burden. Notably, GIV-KO macrophages expressing NOD2-WT displayed a defect similar to GIV-proficient control macrophages expressing the NOD2 *1007fs* variant (Figure 9I). These findings indicate that both GIV and the terminal LRR domain of NOD2 (GIV-binding site on NOD2) are critical for effective NOD2-mediated bacterial clearance.

Collectively, these findings highlight the critical role of the GIV•NOD2 interaction in mediating the protective effects of MDP signaling. Disruption of this interaction, such as in patients harboring the *1007fs* CD-risk variant, may contribute to exaggerated inflammatory responses (via impaired suppression of LPS-induced NF-κB activation) and hinder microbial clearance and restoration of intestinal homeostasis (Figure 9, J and K).

## Discussion

Our study presents 3 major findings. First, we identified a core gene signature that formally defines iColAM and niColAM states in both health and IBD. Within this signature, we established GIV (*CCDC88A* gene) as a critical physical and functional interactor of NOD2, enabling protective and homeostatic NOD2 signaling specifically in noninflammatory macrophage. This protective mechanism, the GIV•NOD2 axis, operates within the lamina propria across models of acute colitis (DSS induced), chronic inflammation (IBD), and acute systemic infection (sepsis). Third, we delineated the molecular basis of the GIV•NOD2 interaction, showing that it is direct, dynamic, and essential for dual antimicrobial and anti-inflammatory macrophage responses to bacterial sensing (Figure 9, J and K). Specifically, this interaction (a) dampens NF-κB–dependent inflammatory signals, and (b) enhances NF-κB–independent pathways that drive PL fusion and bacterial clearance. Together, these dual functions prevent excessive inflammation while ensuring effective microbial control.

Importantly, our findings also reveal a molecular mechanism for the pathogenicity of the high-penetrance CD-associated NOD2 variant, *1007fs*. In the dysbiotic colitic gut, where NOD2 is essential for regulating inflammation and microbial clearance, the inability of GIV to bind the truncated NOD2-*1007fs* variant provides mechanistic insight into how this risk allele contributes to persistent inflammation, dysbiosis, and mucosal pathology. These insights redefine the molecular logic of innate immune sensing and signal integration through NOD2 in intestinal macrophages.

### ColAM signatures provide a computational framework to map macrophage states in the gut.

Our machine learning–assisted analyses identified a subset of genes—ColAMs—from a broader macrophage activation signature (SMaRT; *n* = 338 genes), which reliably distinguish iColAMs from niColAM. The ColAM signature, particularly its 53-gene IBD-associated subset, is clinically relevant and reflects dynamic, disease-relevant macrophage states in transcriptomic datasets.

Notably, our findings show that IBD ColAMs enrich for gut-relevant pathways and successfully resolved macrophage functional states even in bulk RNA-Seq datasets, attesting to their robustness and specificity. In fact, we show that iColAMs and niColAMs dynamically reflect shifts in macrophage function that track with colitis severity—something conventional markers (e.g., CD163) fail to do.

In healthy tissue, niColAMs predominate, likely reflecting the need for tolerogenic surveillance in a microbe-rich environment protected by a single epithelial layer. These macrophages may act as brakes, providing low-grade, tolerogenic surveillance that protects epithelial stem cells and neurons from collateral damage. In contrast, during chronic inflammation and dysbiosis, iColAMs act as accelerators, while niColAMs act as brakes to restrain runaway inflammation. The niColAMs in the setting of colitis appear to transcriptionally resemble M2 macrophages, which have been implicated in mounting an adequate healing response. The niColAMs are induced by NOD2 activation, and GIV appears to be essential for such induction. Disruption of this balance—whether through hyperactive iColAMs or impaired niColAMs (as seen in GIV-KO mice)—may perpetuate inflammation and disease. This “brake and accelerator” framework offers a new conceptual framework for understanding macrophage regulation at mucosal barriers and presents a foundation for therapeutic targeting of macrophage states in IBD.

### GIV enables NOD2 to restrain NF-κB–driven inflammation in noninflammatory macrophages.

Among the 53 IBD-ColAM genes, *CCDC88A* (GIV) emerges as the sole candidate that both physically and functionally interacts with NOD2. While NOD2’s suppression of NF-κB–driven inflammation is well recognized but poorly understood (13), we now show that GIV is essential for this protective function. GIV physically interacts with NOD2, and such binding enables NOD2 to (a) suppress excessive NF-κB activity and (b) drive bacterial clearance via cytoskeletal and PL pathways—both essential for mucosal immunity and homeostasis. In the absence of GIV, NOD2’s antimicrobial and anti-inflammatory functions are impaired, macrophages adopt a reactive phenotype, and host defenses falter. Consequently, macrophages adopt reactive phenotypes, display impaired microbial control, and the host shows heightened susceptibility to colitis and sepsis. Notably, GIV’s inability to bind the NOD2-*1007fs* variant supports a molecular mechanism linking GIV to chronic intestinal inflammation. These data position GIV as a central integrator of gut immune regulation and tissue repair.

These findings build on prior work showing GIV acts as a brake within the LPS/TLR4 signaling cascade (22). GIV’s conserved CT motif binds and dampens inflammatory signaling by TLR1/2, TLR2/6, and TLR3, inducing tolerogenic programs aimed at homeostasis and immunity (22). Thus, GIV emerges as a point of convergence for major PRRs, coordinating tolerogenic responses during microbial sensing (22, 37, 74).

### GIV couples NOD2 to other NF-kB–independent signaling domains and organelle functions.

Our mechanistic analyses show that GIV binds NOD2 via its CT 210 aa, interacting specifically with the terminal LRR repeat of NOD2. This identifies GIV as only the third known protein to directly engage the NOD2-LRR region (75) and 1 of very few to do so in a way that enhances NOD2’s protective, NF-κB–suppressive signaling. While our study defines how GIV shapes NOD2 function, the possibility of reciprocal regulation remains unexplored. It is possible that the GIV•NOD2 interaction may collaborate or compete with GIV-dependent cAMP inhibition (via Gi activation and Gs inhibition) (76) or temporally spatially cross-regulate each other, impacting myriad of inflammatory signals that are shaped by cAMP flux (77–79), including PL fusion events that are critical for microbial clearance (80). This would position NOD2 as another receptor modulating trimeric G-proteins and cAMP through GIV’s CT SLIM motifs, joining a lengthy list of priors (81). Future studies will identify the specific SLIM mediating this interaction and investigate overlap with motifs for TLR4 or Gαi/s binding.

Taken together with its impact on NF-κB–driven inflammation, the NOD2-GIV module likely evolved to balance pathogen elimination with inflammatory restraint. This dual functionality—dampening NF-κB while ensuring efficient PL fusion—may be key to preventing collateral tissue damage during infection and preserving mucosal homeostasis.

### The loss of GIV•NOD2 interaction defines the functional defect in the 1007fs CD-associated variant.

Among the 3 main CD-associated variants (*R702W*, *G908R*, and *1007fs*) that interfere with bacterial recognition (82), *G908R*’s defect lies in impaired MDP contact (72, 73), whereas *R702W* and *1007fs* show defects in palmitoylation and plasma membrane localization (37). Only the *1007fs* variant, which lacks the terminal LRR repeat, fails to regain functionality upon restoring PM localization (83), indicating the *1007fs* variant lacks key functions, perhaps because of the truncated terminal LRR repeat. We show that the *1007fs* variant does not bind GIV and that it lacks the same terminal LRR repeat that is essential for the NOD2•GIV interaction. Because our conclusions are supported by both co-IP and in vitro pull-down assays using recombinant NOD2-LRR proteins, it unlikely that mislocalization artifacts explain the binding loss (as proposed for other NOD2 interactors, e.g., Erbin) (84). Thus, GIV emerges as a first-in-class NOD2-interactor that specifically requires the terminal (10th) LRR repeat—precisely the region lost in the *1007fs* variant. The observation that NOD2-*1007fs*–expressing cells phenocopy GIV-deficient cells, exhibiting heightened inflammation and impaired microbial clearance, further underscores the critical role of this terminal repeat as the GIV-binding site, whose loss disrupts NOD2’s protective signaling.

Because this variant (also termed *3020insC*) is most consistently associated with CD across multiple studies and population groups, and displays 100% disease penetrance, it is not surprising that our GIV-KO animals challenged with *Citrobacter* developed key features of CD: patchy ileocolitis, transmural inflammation, focal muscle hypertrophy, fibrosis, and dysbiosis. Notably, these features arise within just 7 weeks—substantially earlier than the only other known spontaneous murine model of CD SAMP1/YitFcs, which takes approximately 30 weeks (85). Future studies will explore whether GIV-KO mice recapitulate the full molecular and phenotypic spectrum of CD, including defective innate or adaptive immunity and fistula formation.

### Limitations of study.

Although our conclusions are grounded in NOD2-specific phenotypes elicited by MDP stimulation, we lacked tools to directly interrogate the GIV•NOD2 interaction in vivo. Studies will require engineered mutants of GIV and NOD2 that selectively disrupt binding, enabling direct assessment of interaction-dependent functions. Additionally, the observation that MDP enhances GIV•NOD2 binding raises the possibility that other variables—such as pH, ATP levels, or subcellular localization—may modulate this interaction. These contextual factors, known to influence the NOD2 interactome, were not investigated in this study but remain important avenues for future exploration. Finally, we know that GIV can modulate signaling downstream of multiple TLRs, and NOD2 can suppress a subset of those TLRs (15, 17, 22, 86). While this study establishes the role of a functional coupling between GIV and NOD2 in dampening TLR4-driven inflammation, further studies are needed to determine whether GIV-dependent NOD2 signaling broadly suppresses TLR-mediated responses beyond TLR4.

## Methods

Further information can be found in the Supplemental Methods.

### Sex as a biological variable.

All animal experiments used male mice because this study did not assess sex as a biological variable. Male mice were prioritized to ensure continuity with prior sepsis and colitis studies on NOD2 biology. Because neither CD nor the NOD2 1007fs variant shows sex-based susceptibility, and both GIV and NOD2 functions are established across sexes, the core molecular mechanisms are expected to apply broadly.

### Statistics.

All experimental values are presented as the means of replicate experiments ± SEM. Statistical analyses were performed using Prism 9 (GraphPad Software). Differences between the 2 groups were evaluated using AUC classification accuracy, a 2-tailed Student’s *t* test (parametric), and the Mantel-Cox log rank test. To compare more than 3 groups, the unpaired multiple *t* test, 1-way or 2-way ANOVA with Tukey’s multiple comparisons testing was used. *P* < 0.05 was considered significant.

### Study approval.

All mouse studies were approved by the UCSD IACUC (protocol S17223).

### Data availability.

The code related to the computational analyses used in this article is available at https://github.com/sinha7290/NOD2 (commit ID 95101c5e08cde14fec2f6954d112c750a22bfcf9). Mass spectrometry proteomics data have been deposited in the ProteomeXchange Consortium via the Proteomics Identifications (87) partner repository (dataset identifier PXD066180). Newly generated transcriptomic datasets reported in this article have been deposited in NCBI’s Gene Expression Omnibus (GEO) (GSE299285). All other publicly available transcriptomics datasets are accessible through NCBI’s GEO database. All data supporting the findings of this study are included in the Supporting Data Values file. Complete, unedited blots are in the supplemental materials as well as a list of reagents and resources (Supplemental Table 2).

## Author contributions

GDK, MA, and PG conceptualized the project. GDK, MA, SS, MLE, EGM, KCP, STH, JEC, YSM, CRE, MM, and VC were involved in data curation and formal analysis. DTV carried out 16S microbiome analysis. SS, with supervision from PG, carried out all the transcriptomic and proteomic analyses. GDK conducted all animal studies. MSA conducted all biochemical studies. VC assisted GDK in conducting the ELISA. YSM assisted GDK in conducting the qPCR and image analyses. CRE and SRCI performed all molecular biology tasks (construct design, cloning, and mutagenesis). GDK and PG prepared figures for data visualization. PG wrote the original draft; all authors reviewed and edited the manuscript. PG supervised and acquired funding to support the study. All authors approved the final version of the manuscript.

## Funding support

This work is the result of NIH funding, in whole or in part, and is subject to the NIH Public Access Policy. Through acceptance of this federal funding, the NIH has been given a right to make the work publicly available in PubMed Central.

- NIH grants R01-AI141630, UG3TR003355, UG3TR002968, and R01-AI55696 to PG.
- Propel a Cure Foundation and Leona M. and Harry B. Helmsley Charitable Trust.
- American Association of Immunologists Intersect Fellowship Program for Computational Scientists and Immunologists to GDK.
- American Heart Association Career Development Award 24CDA1268506 to GDK.
- American Heart Association Predoctoral Fellowship 25PRE1357971 to MSA.
- The AAI Intersect Fellowship Program to SS.
- NIH grant T32GM8806 to DV.
- NIH postdoctoral fellowship 3R01DK107585-02S1 to SRCI.
- UCSD Agilent Center of Excellence Postdoctoral Fellowship to MM.

## Supplementary Material

Supplemental data

Supplemental data set 1

Supplemental data set 2

Supplemental data set 3

Unedited blot and gel images

Supporting data values

## Version 1 10/02/2025

In-Press Preview

## Version 2 12/01/2025

Electronic publication
