# Supplementary material for: Distinct colitis-associated macrophages drive NOD2-dependent bacterial sensing and gut homeostasis
Source: J Clin Invest. 2025 Oct 2;135(23):e190851. doi: 10.1172/JCI190851 (PMC12646664; doi:10.1172/JCI190851)
Supplement: Supplemental data [file jci-135-190851-s187.pdf]

Distinct Colitis-Associated Macrophages Drive NOD2-Dependent Bacterial Sensing and Gut Homeostasis

Authors: Gajanan D. Katkar[1†], Mahitha Shree Anandachar[1,2†], Stella-Rita Ibeawuchi[2], Ella McLaren[1], Megan Estanol[1], Kennith Carpio-Perkins[1], Shu-Ting Hsu[1], Celia R. Espinoza[1], Jane Coates[1], Yashaswat S. Malhotra[1], Madhubanti Mullick[1], Vanessa Castillo[1], Daniella T. Vo[3], Saptarshi Sinha[1], and Pradipta Ghosh[1,4*]

Affiliations:
[1]Department of Cellular and Molecular Medicine, University of California San Diego, CA, USA.
[2]Department of Pathology, University of California San Diego, CA, USA.
[3]Department of Pediatrics, University of California San Diego, CA, USA.
[4]Department of Medicine, University of California San Diego, CA, USA.

[†] Equal contribution

Conflict of interest statement: Authors have declared that no conflict of interest exists.

KEY WORDS: GIV/Girdin, Guanine-nucleotide exchange modulators (GEMs), CCDC88A, Macrophage, NOD2, MDP, Microbes, Innate immunity

***Correspondence to**:

Pradipta Ghosh, M.D.; Professor, Departments of Medicine, and Cell and Molecular Medicine, University of California San Diego; 9500 Gilman Drive (MC 0651), George E. Palade Bldg, Rm 232, 239; La Jolla, CA 92093. Phone: 858-822-7633: Fax: 858-822-7636: Email: prghosh@ucsd.edu

CATALOG OF SUPPLEMENTARY MATERIALS

1. *Supplementary Methods*
2. *Supplementary Figures and Legends (S1-S4)*
3. *Supplementary Tables (S1-S2)*

SUPPLEMENTARY METHODS

Computational

StepMiner analysis

StepMiner is an algorithm that identifies step-wise transitions using step function in time-series data(1). StepMiner undergoes an adaptive regression scheme to verify the best possible up and down steps based on sum-of-square errors. The steps are placed between time points at the sharpest change between expression levels, which gives us the information about timing of the gene expression-switching event. To fit a step function, the algorithm evaluates all possible steps for each position and computes the average of the values on both sides of a step for the constant segments. An adaptive regression scheme is used that chooses the step positions that minimize the square error with the fitted data. Finally, a regression test statistic is computed as follows:

$$F\ stat = \frac{\sum_{i=1}^{n}(\widehat{X_i} - \bar{X})^2/(m-1)}{\sum_{i=1}^{n}(X_i - \widehat{X_i})^2/(n-m)}$$

Where X_i for $i = 1$ to n are the values, $\widehat{X_i}$ for $i = 1$ to n are fitted values. m is the degrees of freedom used for the adaptive regression analysis. $\bar{X}$ is the average of all the values: $\bar{X} = \frac{1}{n} * \sum_{j=1}^{n} X_j$. For a step position at k, the fitted values $\widehat{X_i}$ are computed by using $\frac{1}{k} * \sum_{j=1}^{n} X_j$ for $i = 1$ to k and $\frac{1}{(n-k)} * \sum_{j=k+1}^{n} X_j$ for $i = k + 1$ to n.

Measurement of classification strength or prediction accuracy:

To measure the strength of classification or prediction accuracy, Receiver Operating Characteristic (ROC) curves were generated for each gene. These curves illustrate the diagnostic ability of a binary classifier system (e.g., high vs. low StepMiner normalized gene expression levels) as its discrimination threshold is adjusted along with the sample order. ROC curves plot the True Positive Rate (TPR) against the False Positive Rate (FPR) at various threshold settings. The Area Under the Curve (AUC) quantifies the probability that a classifier will correctly rank randomly chosen samples into two groups of healthy and IBD patients. Alongside ROC AUC, other classification metrics such as accuracy ((TP + TN)/N; TP: True Positive; TN: True Negative; N: Total Number), precision (TP/(TP+FP); FP: False Positive), recall (TP/(TP+FN); FN: False Negative), and f1 score (2 * (precision * recall)/(precision + recall)) were computed. The Python Scikit-learn package was used to calculate the ROC-AUC values.

Composite gene signature analysis using Boolean Network Explorer (BoNE)

Boolean network explorer (BoNE) (2) provides an integrated platform for the construction, visualization and querying of a gene expression signature underlying a disease or a biological process in three steps: First, the expression levels of all genes in these datasets were converted to binary values (high or low) using the StepMiner algorithm. Second, Gene expression values were normalized according to a modified Z-score approach centered around *StepMiner* threshold (formula = (expr - SThr)/3*stddev). Third, the normalized expression values for every gene were added together to create the final composite score for the gene signature. As a modified Z-score, the composite score of a gene signature ranges from negative to positive values, reflecting the dynamic range of each gene in the signature. These composite scores represent the overall activity or state of biological pathways associated with the genes and, hence, can identify differences between control and query groups *within* any given dataset. However, composite scores cannot be directly compared between different gene signatures within a dataset (as they are not normalized according to the number of genes in each signature), nor can the same signature be compared across datasets (which are individually normalized based on intra-dataset sample distribution or have other inherent differences). The samples were ordered based on the final signature score. Differentially expressed genes were identified using DESeq2 R package. Welch's Two Sample t-test (unpaired, unequal variance (equal_var=False), and unequal sample size) parameters were used to compare the differential signature score in different sample categories. Violin, swarm and bubble plots are created using python seaborn package version 0.10.1. Pathway enrichment analyses for genes were carried out using the KEGG database (3). Violin plots are created using python seaborn package version 0.10.1.

Correlation Heatmap

The correlation coefficient between two gene expression values in a transcriptomics dataset was calculated by plotting them in a scatter plot and measuring the correlation coefficient using Python's SciPy library.

Bulk RNAseq Deconvolution

The *in-silico* deconvolution of bulk RNA sequencing data to estimate immune cell-type abundance in murine colon samples was performed using the Granulator R package (4). To normalize cell-type abundances, we utilized the immune cell signature matrix developed by Monaco et al., (5).

Experimental

Cell culture

Thioglycolate-elicited murine peritoneal macrophages (TGPMs) were collected from peritoneal lavage of 8- to 12-wk-old C57BL/6 mice with ice cold RPMI (10 ml per mouse) 4 days after intraperitoneal injection of 3 ml of aged, sterile 3% thioglycolate broth (BD Difco, USA) and cultured as described previously (6). Cells were passed through 70 μm filter to remove possible tissue debris contamination during harvesting. Cells were counted, centrifuged, and resuspended in RPMI-1640 containing 10 % FBS and 1% penicillin/streptomycin. Cells were plated with required cell density and the media was changed after 4 h to remove non adherent cells. Cells were allowed to adjust to overnight culture before the addition of stimuli as indicated in the Figure legends. RAW264.7, HEK293T and HeLa cells (from ATCC) were maintained in the DMEM media containing 10% FBS and 1% penicillin/streptomycin. THP1 reporter cells (THP1-Dual™ Cells, InVivoGen, USA) derived from THP-1, a human monocytic cell line and parental THP1 WT cells were maintained RPMI media containing 10% FBS and 1% penicillin/streptomycin.

Bacteria and bacterial culture

Citrobacter rodentium (strain DBS100) and Adherent Invasive *Escherichia coli* strain LF82 (AIEC-LF82) were cultured from a single colony inoculation into LB broth for 6-8 h on shaking incubator, followed by overnight culture under oxygen-limiting conditions, without shaking, to maintain their pathogenicity as done previously (7-9). Bacterial cells were counted by measuring an absorbance at 600 nm (OD600), washed with PBS, and infected with indicated MOI in Figure legends.

Mice

Ccdc88a$^{fl/fl}$ mice were a gift from Dr. Masahide Takahashi (Nagoya University, Japan) and was developed as described (10). LysM$^{Cre/Cre}$ mice (B6.129P2-Lyz2t (5) (cre)Ifo/j) were purchased from the Jackson Laboratory. *Ccdc88a*$^{fl/fl}$ x LysM$^{Cre/-}$ mice were generated previously by us as described (11) and were maintained as homozygous floxed and heterozygous LysMcre. Primers required for genotyping are mentioned in **Supplementary Table 2**. Both male and female mice (8-12 weeks) were used and maintained in an institutional animal care at the University of California San Diego animal facility on a 12-hour/12-hour light/dark cycle

(humidity 30–70% and room temperature controlled between 68–75 °F) with free access to normal chow food and water. All mice studies were approved by the University of California, San Diego Institutional Animal Care and Use Committee (IACUC).

Fecal pellet collection, DNA extraction and 16S rRNA sequencing

Individual mice fecal pellets from co-housed GIV-KO mice and their littermate WT controls (8-12 weeks) were collected in clean containers and frozen at -80ºC untill use. Samples were transported to microbiome core facility, University of California, San Diego for 16S rRNA processing. Total DNA was extracted from the individual mice fecal pellets using MagMAX Microbiome Ultra Nucleic Acid Isolation kit, (Thermo Fisher Scientific, USA) and automated on KingFisher Flex robots (Thermo Fisher Scientific, USA). 16S rRNA gene amplification was performed according to the Earth Microbiome Project protocol (12). Briefly, Illumina primers with unique forward primer barcodes (13) were used to amplify the V4 region of the 16S rRNA gene (515F-806R (14)) with single reactions per sample (15). Equal volumes of each amplicon were pooled, and the library was sequenced on the Illumina MiSeq sequencing platform with paired-end 150 bp cycles.

16S rRNA gene data analysis

QIIME2 (16) was used to process the demultiplexed files. Sequences were filtered, denoised and trimmed to 150 bp using DADA2 (17), which is used to correct Illumina-sequenced amplicon errors. The sequences were classified using the q2-feature-classifier plugin from QIIME2 that was trained on the Green genes 13_5 99% OTUs trimmed to 150 bp. Alpha and beta diversity plots were generated using the R microbiome package (18). Alpha diversity was estimated using Faith's phylogenetic diversity and Shannon diversity. Beta diversity was estimated using Non-Metric Multidimensional Scaling (NMDS) and Bray-Curtis dissimilarity (18).

***C. rodentium* induced infectious colitis**

C. rodentium (strain DBS100) induced infectious colitis studies were performed on 8-week-*old* GIV-KO and their littermate WT control mice. *C. rodentium* were grown overnight in LB broth with shaking at 37 ºC. Mice were gavaged orally with 5×10^8 CFU in 0.1 ml of PBS (19, 20). To determine viable bacterial numbers in faeces, fecal pellets were collected from individual mice, homogenized in ice-cold PBS, serially diluted, and plated on

MacConkey agar plates. Number of CFU was determined after overnight incubation at 37 ºC. Colon samples were collected to assess histology in the 7th week.

DSS-induced colitis

To induce colitis, mice were fed with drinking water containing 2.5% dextran sulfate sodium (DSS, w/v) (MP Biomedicals, MW 36–50 kDa) for five days and then replaced normal drinking water as described (21, 22). For treatment study, MDP (100 mg/mouse/day) was administered via intraperitoneal route in 100 ml total volume sterile saline every alternate day starting from day 0 of experiment. Mice were sacrificed on the 14th day, and colon length was assessed. Colon samples were collected to assess the levels of mRNA (by qPCR). Drinking water levels were monitored to determine the volume of water consumption. Weight loss, stool consistency, and fecal blood were recorded fro individual animals, and these parameters were used to calculate an average Disease Activity Index (DAI) as described previously (23). Colon histology was assessed in hematoxylin and eosin-stained tissue sections using standard protocols.

***E. coli*-induced sepsis**

E. coli (strain RS218) was grown overnight in Lysogeny broth (LB) media with shaking at 37ºC. Next morning, fresh LB media was used to dilute cultures to 1:50 and grow up to mid-log phase, washed twice with PBS, and reconstituted in PBS. GIV-KO mice (8–12-week-old) and their control WT littermates were injected with 1.5×10^8 CFU of *E. coli* in 200 ul and mice survival was recorded for 24h post-infection. Mice were pre-treated with MDP (100 µg i.p.) or PBS 18 h before the infection.

Transmission electron microscopy (TEM) and immunogold EM staining

Cells were fixed with Glutaraldehyde in 0.1M Sodium Cacodylate Buffer, (pH 7.4) and post-fixed with 1% OsO_4 in 0.1 M cacodylate buffer for 1 hr on ice. The cells were stained with 2% uranyl acetate for 1 hr on ice and dehydrated in graded series of ethanol (50-100%) while remaining on ice. The cells were washed once with 100% ethanol and twice with acetone (10 min each) and embedded with Durcupan. Sections were cut at 60 nm on a Leica UCT ultramicrotome and picked up on 300 mesh copper grids. All sections were post-stained sequentially 5 min with 2% uranyl acetate and 1 min with Sato's lead stain. Samples were visualised using JEOL

1400 plus equipped with a bottom-mount Gatan OneView (4k x 4k) camera. For immunogold staining to determine co-localization of NOD2 and GIV. The sections were incubated with mouse anti-NOD2 antibody (Santa Cruz, sc-56168, 1:50 dilution) and rabbit anti-GIV antibody (Millipore sigma, ABT80; 1:50 dilution) followed by secondary antibodies 18 nm colloidal gold of donkey anti-rabbit IgG and 12 nm gold of donkey anti-mouse IgG (Jackson ImmunoResearch Laboratories, Inc.). Samples were visualised using JEOL 1400 plus equipped with a bottom-mount Gatan OneView (4k x 4k) camera.

Plasmid constructs

The plasmids used in this study were HA-Nod1 and HA-Nod2 (from Dana Philpott (24) and M. D'Amato (25)); and His-Myc tagged NOD2 constructs (His-myc-Nod2 FL, ΔCARD-His-myc-Nod2, ΔNBD-His-myc-Nod2, ΔLRR-His-myc-Nod2) were generous gift from Santanu Bose (26), . All other Nod2 mutants were generated by site directed mutagenesis. GST-Nod2-LRR and Myc-Nod2 were cloned into pGEX-6P and pCMV-myc vectors, respectively, using cloning sites indicated in **Supplementary Table 2**.

Protein expression and purification

GST and His-tagged proteins were expressed in *E. coli* strain BL21 (DE3) and proteins were purified as described (11, 27, 28). Briefly, to stimulate protein expression, bacteria cultures were activated with 1 mM IPTG overnight at 25°C. Bacteria were then pelleted and resuspended in either GST lysis buffer (25 mM Tris-HCL (pH 7.4), 20 mM NaCl, 1 mM EDTA, 20% (vol/vol) glycerol, 1% (vol/vol) Triton X-100, protease inhibitor cocktail) or His lysis buffer (50 mM NaH2PO4 (pH7.4), 300 mM NaCl, 10 mM imidazole, 1% (vol/vol) Triton-X-100, protease inhibitor cocktail), sonicated and lysates were cleared by centrifugation at 12,000 x g at 4°C for 30 mins. Supernatant was then affinity purified using glutathione-Sepharose 4B beads or HisPur Cobalt Resin, followed by elution, overnight dialysis in PBS, and then stored at -80°C until use.

Transfection, lysis, and quantitative immunoblotting

HEK293T and HeLa cells were cultured in DMEM media containing 10% FBS and antibiotics according to the ATCC guidelines. Cells were transfected using polyethylenimine for DNA plasmids following the manufacturers' protocols. Lysates for immunoprecipitation assays were prepared by resuspending cells in lysis buffer (20 mM HEPES, pH 7.2, 5 mM Mg-acetate, 125 mM K-acetate, 0.4% Triton X-100, 1 mM DTT) supplemented with 500

μM sodium orthovanadate, phosphatase inhibitor cocktails (Sigma) and protease inhibitor cocktails (Roche), and cleared (10,000 x *g* for 10 min) before use. For immunoblotting, proteins were fractionated by SDS-PAGE and transferred to PVDF membranes (Millipore). Membranes were blocked with 5% nonfat milk dissolved in PBS before incubation with primary antibodies followed by detection with secondary antibodies using infrared imaging with two-color detection and quantification were performed using a Li-Cor Odyssey imaging system. All Odyssey images were processed using Image J software (NIH) and assembled for presentation using Photoshop and Illustrator software (Adobe).

Immunoprecipitation, *in vitro* GST-pulldown with recombinant purified protein or cell lysates

For *in vitro* pulldown assays, purified GST-tagged proteins from *E. coli* were immobilized onto glutathione Sepharose beads by incubating with binding buffer (50 mM Tris-HCl (pH 7.4), 100 mM NaCl, 0.4% (vol/vol) Nonidet P-40, 10 mM MgCl2, 5 mM EDTA, 2 mM DTT) overnight at 4°C with continuous rotation. GST-protein bound beads were washed and incubated with purified His-tagged proteins resuspended in binding buffer or with pre-cleared (by centrifugation at 10,000xg for 10 min) cell lysates prepared using lysis buffer [20 mM HEPES, pH 7.2, 5 mM Mg-acetate, 125 mM K-acetate, 0.4% Triton X-100, 1 mM DTT, 500 μM sodium orthovanadate supplemented with phosphatase inhibitor cocktail (Sigma Aldrich) and protease inhibitor cocktail (Roche)] for 4 hrs at 4°C. After binding, bound complexes were washed four times with 1 ml phosphate wash buffer (4.3 mM Na2 HPO4, 1.4 mM KH2PO4 (pH 7.4), 137 mM NaCl, 2.7 mM KCl, 0.1% (vol/vol) Tween-20, 10 mM MgCl2, 5 mM EDTA, 2 mM DTT, 0.5 mM sodium orthovanadate) and eluted by boiling in Laemmli buffer (5% SDS, 156 mM Tris-Base, 25% glycerol, 0.025% bromophenol blue, 25% β-mercaptoethanol).

Co-immunoprecipitation assays

N-terminal HA- or Myc-tagged NOD2 and C-terminal FLAG-tagged GIV full length proteins were co-expressed in HEK293T cells and 48 h after transfection, cells were stimulated with 10 μg/ml MDP (L18-MDP, Invivogen) for 1, 3 and 6 h followed by cell lysis in lysis buffer (20 mM HEPES, pH 7.2, 5 mM Mg-acetate, 125 mM K-acetate, 0.4% Triton X-100, 1 mM DTT, 0.5 mM sodium orthovanadate, Tyr phosphatase inhibitor cocktail, Ser/Thr phosphatase inhibitor cocktail, and protease inhibitor cocktail). For immunoprecipitation, equal aliquots of clarified cell lysates were incubated for 3h at 4°C with 2 μg of appropriate antibody [either anti-HA mAb or anti-

FLAG M2Ab (Sigma Monoclonal ANTI-FLAG® M2, Clone M2)]. Subsequently, protein G Sepharose beads (GE Healthcare; 40 µl 50% v:v slurry) were added and incubated at 4°C for an additional 60 min. Beads were washed 4 times (1 ml volume each wash) in PBS-T buffer [4.3 mM Na2HPO4, 1.4 mM KH2PO4, pH 7.4, 137 mM NaCl, 2.7 mM KCl, 0.1% (v:v) Tween 20, 10 mM MgCl2, 5 mM EDTA, 2 mM DTT, 0.5 mM sodium orthovanadate] and immune complexes were eluted by boiling in Laemmli's sample buffer. Bound immune complexes were separated on SDS PAGE and analyzed by immunoblotting with anti-HA, anti-Myc and anti-FLAG antibodies. In assays where the impact of nucleotides was studied on protein-protein interactions, cells were were first permeabilized with 4 µg/ml of streptolysin-O (Sigma-Aldrich, Budapest, Hungary) for 30 min at 37°C followed by incubation with nucleotides (ADP, ATP, or ATPgS).

Proximity ligation assay (PLA)

THP1 cells grown on glass coverslips nearly 70% confluency, washed two times with PBS, and fixed 20 min at room temperature with 4% (wt/vol) PFA. After washing with PBS, cells were processed for PLA using the Duolink *In Situ* PLA kit according to the manufacturer's instructions. Briefly, coverslips were blocked for 1 hour at room temperature, followed by a 1-hour incubation with primary antibodies targeting GIV and NOD2. Subsequently, cells were incubated with PLA probes for 1 hour in a humidified chamber at 37°C. If the two oligo probes are in proximity, ligation occurs, and further amplification steps enable fluorescence detection of the reaction as bright red dots (566–594 nm). Cells were then counterstained with DAPI to visualize nuclei and imaged using Cytation10 widefield microscopy or leica stellaris confocal. Quantification of PLA dots were performed using Gen5 or ImageJ software.

Confocal immunofluorescence

Cells were fixed with 4% paraformaldehyde in PBS for 30 min at room temperature, treated with 0.1 M glycine for 10 min, and subsequently blocked/permeabilized with blocking buffer (PBS containing 1% BSA and 0.1% Triton X-100) for 20 min at room temperature. Primary and secondary antibodies were incubated for 1 h at room temperature or overnight in blocking buffer. Dilutions of antibodies used were as follows: HA (1:200) and DAPI (1:1000). Alexa fluor fluorescent dye conjugated secondary antibodies were used at 1:500 dilutions. For visualizing actin organizations cells were stained with 16 mM Phalloidin Alexa Fluor^{TM}-594 for 30 min at room

temperature, washed three times with PBS. ProLong Glass antifade reagent is used for mounting the coverslips on glass slides.

Immunofluorescence staining of colon tissue sections

Mouse colon Swiss rolls were fixed in zinc paraformaldehyde to prepare FFPE tissue blocks. FFPE tissue sections of 4 μm thickness underwent deparaffinization and rehydration. Heat-induced epitope retrieval was performed using Tris-EDTA buffer (pH 9.0) in a pressure cooker. Tissue sections were blocked using 2.5% goat serum, followed by incubation with primary antibody overnight in a humidified chamber at 4°C. Primary antibodies used for immunostaining were CD68 (mouse monoclonal, dilution 1:150, Abcam ab201340) and CD163 (rabbit polyclonal, dilution 1:150, Abcam ab182422). Goat anti-mouse IgG (H+L) AlexaFluor488 and Goat anti-rabbit (H+L) Alexa Fluor 594 were used as secondary antibodies (dilution 1:500) in combination with DAPI (dilution 1:1000) staining. Images were acquired on a Stellaris 5 Confocal Microscope (Leica microsystems) and images were analyzed using the software package QuPath (Version: 0.5.0) and further processed using Fiji/ImageJ software (NIH, Bethesda, USA).

CD68 and CD163 quantification

IF images were obtained in a random, genotype-blinded fashion and analyzed using QuPath (Version: 0.5.0). For each image, nuclei were detected based on DAPI and cell boundaries were drawn using a uniform 5um expansion with the QuPath built-in cell detection feature. An object classifier was created for the identification of CD163+, CD68+, and negative cell populations. Percent positivity was calculated by dividing the number of positive cells by the total number of cells in each image. Welch's two sample unpaired t-test was performed to compute p values.

NFκB reporter assay

RAW 264.7 cells (50000 cells/well in 96-well plate) were transfected together with 50 ng NFκB reporter plasmid and 5 ng Renilla luciferase control plasmid. After 24h, cells were stimulated with L18-MPD (10 μg/ml) for 6 hr and NFκB activity was assessed using the Dual-luciferase Reporter Assay System using manufacturers protocol. For HeLa cells 10000 cells/well in 96-well plate were seeded and transfected with 25 ng NFκB reporter plasmid,

0.5 ng Renilla luciferase control plasmid and either 5 ng/well of NOD2-WT or its mutants. Cells were primed with or without L18-MPD (10 μg/ml) for 16 hr before stimulating with LPS100ng/ml for 6h and NFκB activity was assessed using a noncommercial dual luciferase enzyme assay as described (29).

RNA extraction and Quantitative PCR

Total RNA was isolated using TRIzol reagent (Life Technologies) and Quick-RNA MiniPrep Kit (Zymo Research, USA) as per manufacturer's guidelines. RNA was converted into cDNA using the qScript™ cDNA SuperMix (Quantabio) and quantitative RT-PCR (qPCR) was carried out using PowerUp™ SYBR™ green master mix (Applied Biosystems, USA) with the StepOnePlus Quantitative platform (Life Technologies, USA). The cycle threshold (Ct) of target genes was normalized to 18S rRNA gene and the fold change in the mRNA expression was determined using the 2^-ΔΔCt method.

Gentamicin Protection Assay

Gentamicin protection assay was used to quantify viable intracellular bacteria as described previously(7). Approximately, 2×10^5 TGPMs were seeded into 12-well culture dishes overnight before infection at an MOI of 10 for 1 h in antibiotic-free RPMI media containing 10% FBS in a 37 °C CO2 incubator. Cells were then incubated with gentamicin (200 μg/ml) for 90 min after PBS was to kill extracellular bacteria. After incubation time cells were washed with PBS and subsequently lysed in 1% Triton-X 100, lysates were serially diluted and plated on LB agar plates. Bacteria colonies (CFU) were counted after overnight (16 h) incubation at 37 °C. To test effect of MDP, cells were pre-treated overnight with MDP (10 μg/ml).

Cytokine Assays

Cytokines including TNFA, IL6, IL1B and IL10 were measured in cell supernatant using ELISA MAX Deluxe kits from Biolegend as per manufacturer's protocol.

SUPPLEMENTARY FIGURES

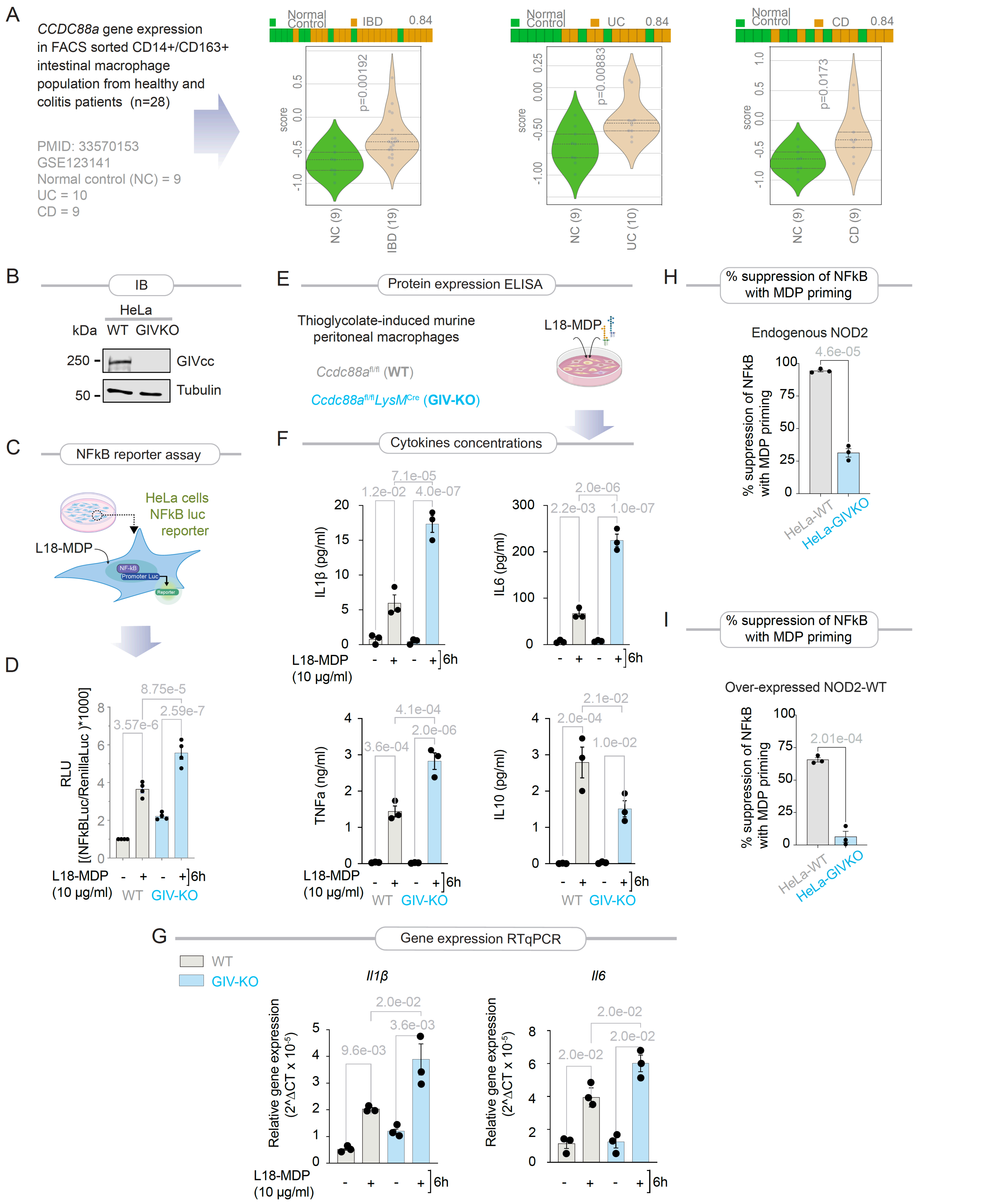

Supplementary Figure 1: GIV is required for MDP-stimulated inflammatory resolution. Related to **Figures 1, 2 and 5**.

A. Violin plots display *CCDC88A* expression in macrophages that were isolated from the lamina propria of colons from healthy and IBD (UC and CD) subjects. Bar plots on top denote sample classification accuracy using *CCDC88A* as single gene expression score and the numbers on top indicate ROC AUC.

B. Immunoblot (IB) of control (HeLa-WT) or GIV-KO (HeLa-GIV-KO) HeLa cells confirming depletion of GIV by >95%.

C-D. Schematics (C) display the NFkB reporter assay in HeLa cells and bar graphs (D) display the fold change in NFkB activity.

E-F. Schematic (E) of study design to assess cytokines produced by peritoneal macrophage stimulated with L18-MDP (10 µg/ml) for 6 h. Bar graphs (F) display the concentrations of the indicated cytokines, as determined by ELISA.

G. Bar graphs display the gene expression in cell lysates in E, as determined by RT qPCR.

H-I. Bar graphs display the fold change in NFkB activity induced by LPS (100 ng/ml) for 6h, following 24-hour priming with L18-MDP (10 µg/ml) in HeLa cells. Panel H represents cells with endogenous NOD2, while Panel I represents cells with overexpressed NOD2.

Statistics: All results are displayed as mean ± SEM (n=3 biological replicates). Statistical significance was tested using one-way ANOVA with Tukey's test (F and G), Two-tailed Student's t test (H and I) and Welch's t-test (A). *p*-value ≤ 0.05 is considered as significant.

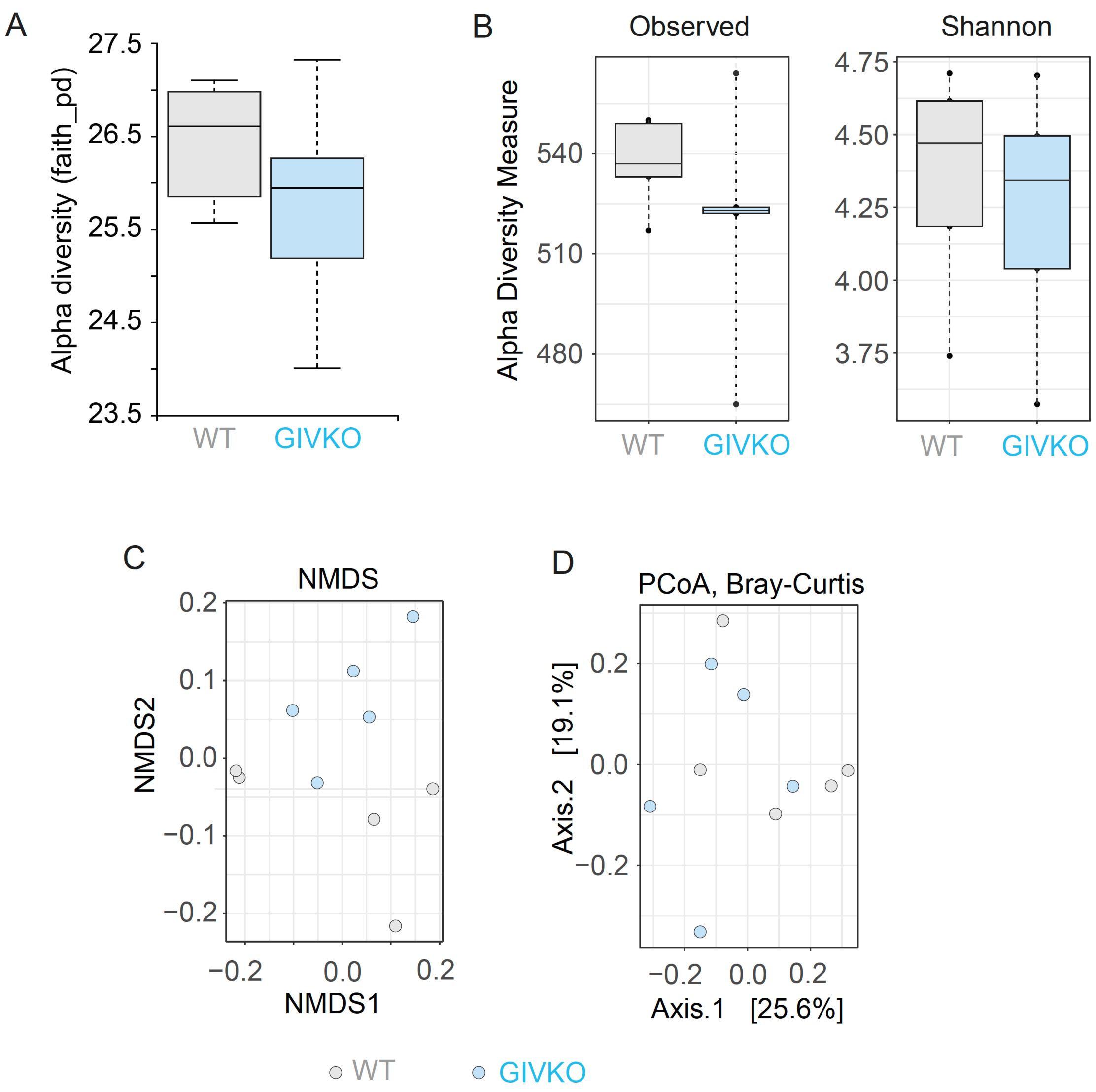

Supplementary Figure 2: Fecal microbiome analysis confirms spontaneous dysbiosis in myeloid-specific (LysMCre) GIV-KO mice age 8-12 wk. Related to **Figure 4A**.

A-B. Box plots display alpha diversity indices (Observed and Shannon), of fecal microbiome communities within GIV-KO and their littermate controls.

C. Non-Metric Multidimensional Scaling (NMDS) ordination of fecal microbiome communities within GIV-KO and their littermate controls

D. PCoA plot of beta diversity of fecal microbiome communities within GIV-KO and their littermate controls.

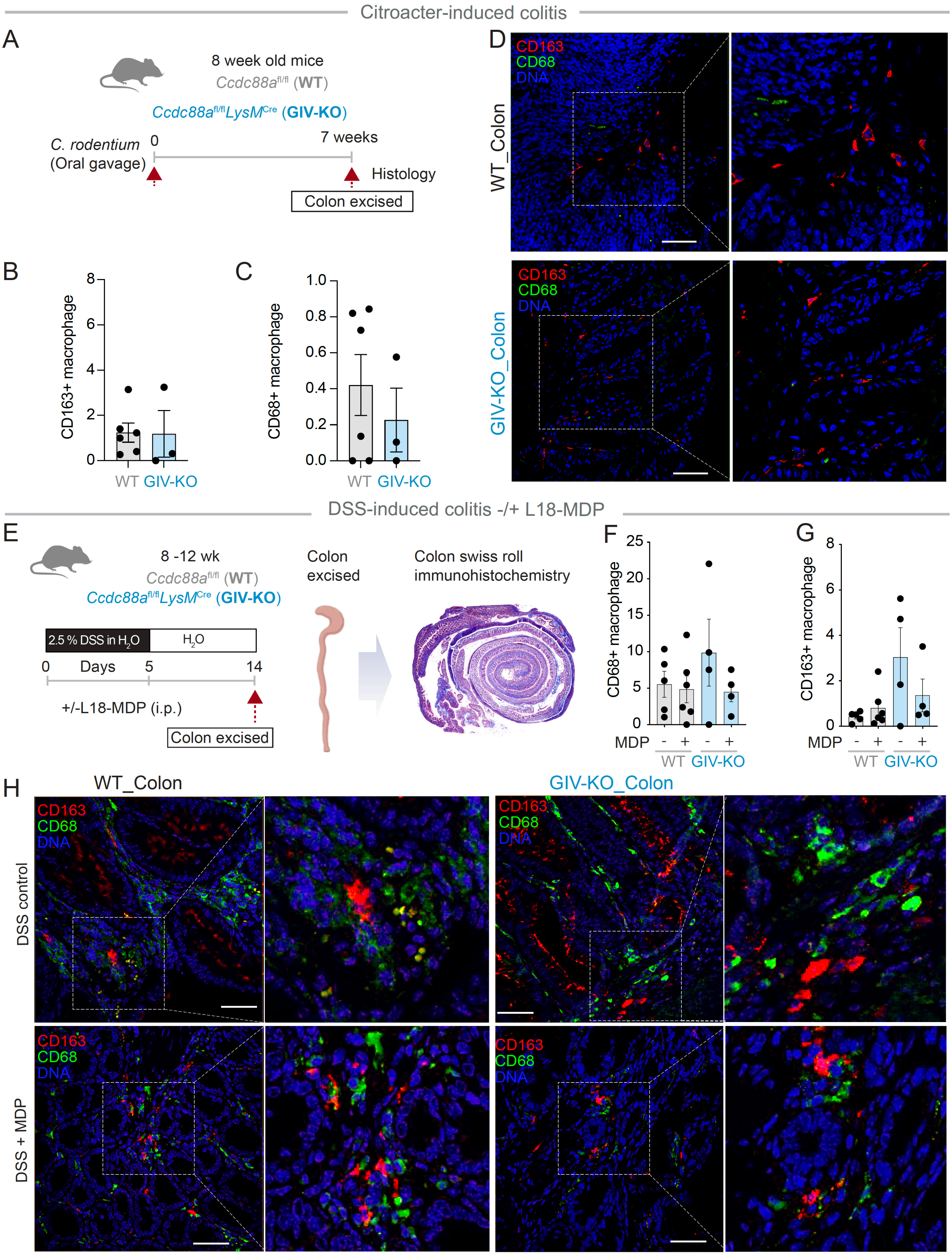

Supplementary Figure 3: The Loss of GIV does not impede macrophage recruitment and expression of conventional markers for polarization. Related to **Figures 4-5**.

A-D. Panels describing the experimental design (**A**) and macrophage population distribution in the colon (**B-D**) in an infectious colitis model of GIV-KO and control littermates induced using *Citrobacter rodentium* (initially termed *Citrobacter freundii* biotype 4280 (30); strain name DBS100; 5 x 10^8 CFU/200ul/mouse. GIV-KO, n=8; WT, n=6. Findings are representative of two independent repeats. *See Figure 4C-I for the details*.

B-C. Quantification of $CD163^+$ (B) and $CD68^+$ (C) macrophages in colonic sections. n=4-6. **D.** Representative immunofluorescence images showing CD163 (red), CD68 (green), and nuclei (blue) in colon tissues. Scale bars = 50 µm.

E–H. Schematic (E) displays the study design for DSS-induced colitis. GIV-KO, n=5; WT, n=5. Findings are representative of two independent repeats. *See Figure 5A-E for the details.*
(F–G) Quantification of $CD68^+$ (F) and $CD163^+$ (G) macrophages in colon tissues. n = 5–6 mice per group.
(H) Representative immunofluorescence images of colon sections from DSS-only and DSS+MDP groups showing CD163 (red), CD68 (green), and nuclei (blue). Scale bars = 50 µm.

All graphs represent mean ± SEM. Statistical comparisons were made using one-way ANOVA with Tukey's test (F, G) and two-tailed Student's t-test (B, C). *p*-value ≤ 0.05 is considered as significant.

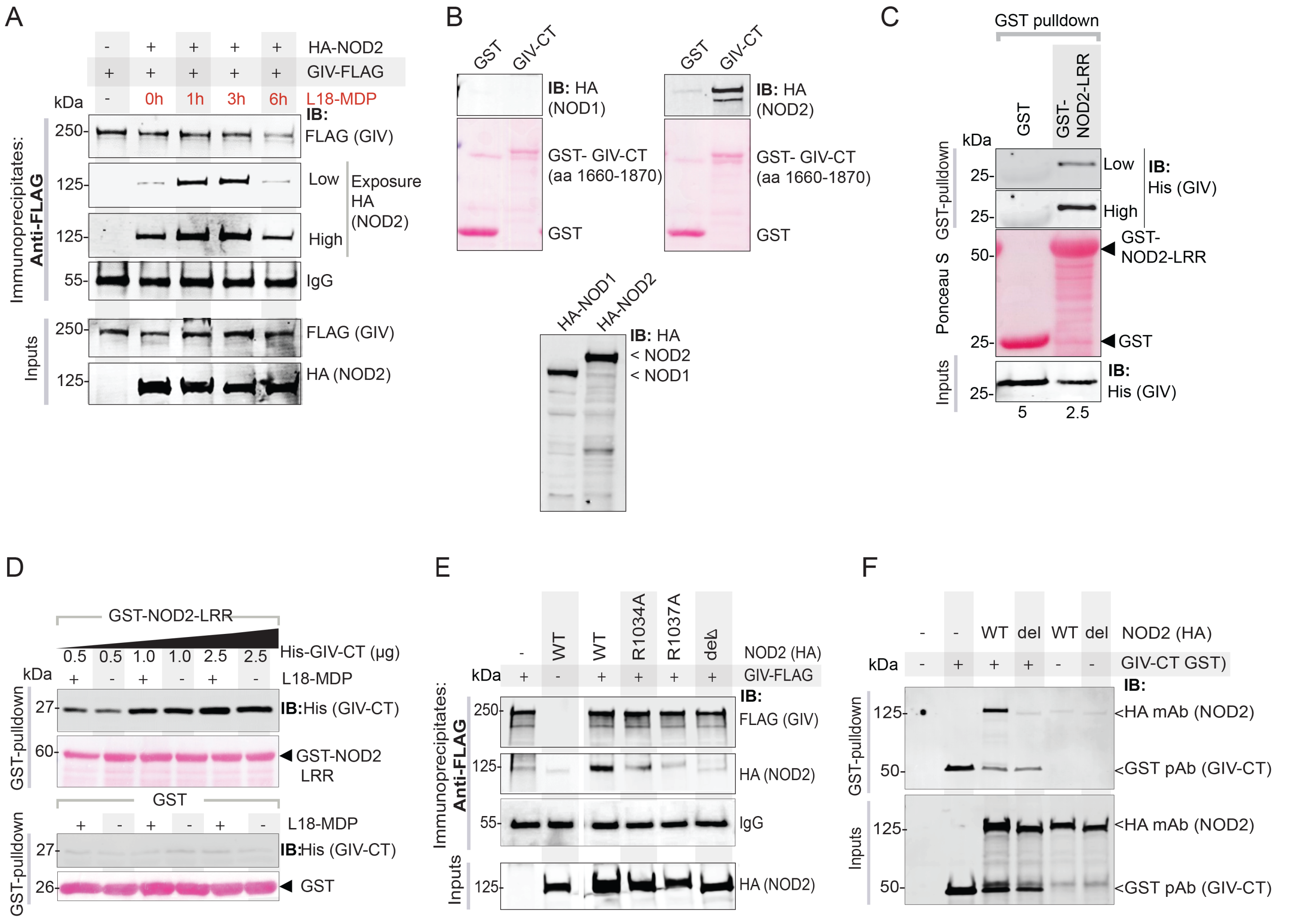

Supplementary Figure 4: The NOD2(LRR)•GIV(C-term) interaction is a dynamic. Related to **Figures 7-8**.

A. FLAG-tagged GIV was immunoprecipitated with anti-FLAG mAb from equal aliquots of lysates of HEK cells expressing GIV-FLAG and HA-NOD2, stimulated (+) or not (-) with L18-MDP (10 µg/ml) for indicated time points. Immunoprecipitated (IP; top) complexes and input (bottom) lysates were analyzed for NOD2 and GIV by immunoblotting (IB).

B. Lysates of HEK cells expressing HA-tagged NOD1/2 proteins were used in a GST pulldown assay with GST or GST-GIV-CT immobilized on Glutathione Sepharose beads. Bound NOD1/2 proteins were visualized by immunoblotting (IB).

C. Recombinant His-GIV-CT proteins were used in a GST pulldown assay with GST or GST-NOD2-LRR immobilized on Glutathione Sepharose beads. Bound GIV were visualized by immunoblotting (IB).

D. Recombinant His-GIV-CT proteins (0.5, 1, 2.5 µg) were used in a GST pulldown assay with GST or GST-NOD2-LRR immobilized on Glutathione Sepharose beads, in presence (+) or absence (-) of 10x molar excess of L18-MDP. If MDP and GIV share the same binding site on the LRR domain of NOD2, excess of MDP is expected to compete with GIV and reduce its binding to NOD2-LRR. Bound GIV was visualized by immunoblotting (IB).

E. FLAG-tagged GIV was immunoprecipitated with anti-FLAG mAb from equal aliquots of lysates of HEK cells expressing GIV-FLAG and either wild-type (WT) or mutant HA-NOD2 constructs. Immunoprecipitated (IP; top) complexes and input (bottom) lysates were analyzed for NOD2 by immunoblotting (IB).

F. GST-GIV-CT was pulled down using Glutathione Sepharose beads from equal aliquots of lysates of HEK lysates expressing the wild-type (WT) or del mutant HA-NOD2 construct either alone (last two lanes) or with GST-GIV-CT (aa 1660-1870; mammalian p-CEFL vector). Bound NOD2 proteins and similar expression of GIV-CT was assessed by immunoblotting (IB) using anti-HA (NOD2) and anti-GST (GIV-CT) antibodies.

SUPPLEMENTARY TABLES

Supplementary Table 1: NOD2 mutations and its clinical relevance in CD patients.

Mutation	Percentage of CD Patients	% CD diagnosis	Mechanistic insights	Variation Caused	Phenotypic Consequence
**1007fs (Frameshift Stop codon mutation)**	31%	100%	Impaired palmitoylation (31), and protein mislocalization<br><br>Forced PM-localization of protein *does not* restore function (32).<br><br>MDP-binding site unaffected<br><br>Loss of binding to GIV (current work)	- Mutant is unable to recognize MDP to initiate NFkB activation<br>- Associated defective release of IL-10 from blood mononuclear cells (PBMC) after stimulation with the TLR2 ligands, PGN and Pam3Cys-KKKK, and LPS.	- The genotype relative risk (GRR) for developing CD in heterozygotes and homozygotes of this mutation alone is 3.29 ±0.64 and 34.66 ±12.87 respectively<br><br>- Homozygosity is strongly associated with gastroduodenal CD and younger age at diagnosis<br><br>- Homozygous patients demonstrate a much more severe disease phenotype than other patients with Crohn's disease and have an increased risk for ileal stenoses and surgical interventions
**R702W (Missense Substitution Mutation)**	32%	100%	Impaired palmitoylation (31), and protein mislocalization<br><br>Forced PM-localization of protein restores function (32).<br><br>MDP-binding site unaffected<br><br>Loss of binding to GIV (current work)	- Monocyte-derived dendritic cells (MoDCs) produced significantly higher levels of IL-12 on stimulation with whole bacteria<br><br>- MoDCs carrying the mutation displayed an increased basal level of IL-8 release, which, after a bacterial encounter, equilibrated to the levels similar to healthy controls.	- GRR for developing CD in heterozygotes and homozygotes of this mutation alone is 1.97±0.85 and roughly 3.05 respectively<br><br>- Positive independent association with structuring behavior and granuloma formation
**G908R (Missense Substitution Mutation)**	18%	80%	Normal palmitoylation (31) and protein mislocalization.<br><br>Forced PM-localization of protein restores function (32).<br><br>One of the residues that form contact site for MDP and hence, MDP recognition could be directly impacted (33).<br>GIV binding (current work).	-MoDCs produced significantly higher levels of IL-12 on stimulation with whole bacteria	- GRR for developing CD in heterozygotes and homozygotes of this mutation alone is 1.97±0.85 and 4.55±1.34 respectively

Supplementary Table 2: List of reagents and resources

REAGENT or RESOURCE	SOURCE	IDENTIFIER
Biological Samples and Cell Lines		
**Adherent-invasive *E. coli* LF82 (*AIEC*-LF82)**	Prof. Arlette Darfeuille-Michaud	(34)
***E. coli* (strain RS218)**	Prof. Victor Nizet	(11)
Citrobacter rodentium (strain DBS100)	ATCC (35)	
RAW 264.7	ATCC	TIB-71
shControl RAW 264.7	(11)	
shGIV RAW 264.7	(11)	
HEK293T	ATCC	ATCC® CRL-11268
HeLa parental	ATCC	ATCC® CCL-2
HeLa GIV KO (CRISPR Cas9)	(36)	n/a
LysMcre mice (B6.129P2-Lyz2$^{tm1(cre)Ifo}$/j)	The Jackson Laboratory	004781
Girdin flox mice	Asai et al. Biochem. Biophys. Res. Commun., 2012	N/A
Ccdc88a$^{fl/fl}$ LysM$^{Cre/-}$ mice	(11)	
Chemicals and Reagents		
L18-MDP	Invivogen	
Lipopolysaccharide (E. coli O111:B4)	Sigma-Aldrich	L4391
PowerUpä SYBR^{ä} Green Master Mix	Applied Biosciences	A25741
qScript^{ä} cDNA SuperMix	QuantaBio	101414
Direct-zol RNA Miniprep Kit	Zymo Research	R1051
TRIzol^{ä} Reagent	Invitrogen	15596018
ELISA MAX^{ä} Deluxe Set Mouse IL-6	BioLegend	431304
ELISA MAX^{ä} Deluxe Set Mouse IL-1b	BioLegend	432604
ELISA MAX^{ä} Deluxe Set Mouse IL-10	BioLegend	431414
ELISA MAX^{ä} Deluxe Set Mouse TNF-a	BioLegend	
Dextran Sulfate Sodium Salt (Colitis Grade)	MP Biomedicals, LLC	160110
Hemoccult II	Beckman Coulter	61130
Anti-CD68 (mouse monoclonal, dilution 1:150)	Abcam	*ab201340*
Anti-CD163 (rabbit polyclonal, dilution 1:150)	Abcam	*ab182422*
Goat anti-mouse IgG (H+L) AlexaFluor488 (dilution 1:500)	Invitrogen	*Cat # A-11001*
Goat anti-rabbit (H+L) Alexa Fluor 594 (dilution 1:500)	Invitrogen	*Cat # A-11012*

Rabbit anti-GST	Cell signaling Technologies	*Cat # 2625*
Rabbit anti-HA	Proteintech	*Cat# 51064-2-AP*
Zinc Formalin Fixative	Sigma-Aldrich	Z2902
Primers		
Species/Targets	Forward primer (5'→ 3')	Reverse primer (3'→ 5')
Mouse IL-6 qPCR primers	TGGAGTCACAGAAGGAGTGGCTAAG	TCTGACCACAGTGAGGAATGTCCAC
Mouse IL-1b qPCR primers	GCCTTGGGCCTCAAAGGAAAGAATC	GGAAGACACAGATTCCATGGTGAAG
Mouse TNFA qPCR primers	ATAGCTCCCAGAAAAGCAAGC	CACCCCGAAGTTCAGTAGACA
Mouse IL-10 qPCR primers	CCCTGGGTGAGAAGCTGAAG	CACTGCCTTGCTCTTATTTTCACA
Mouse 18S qPCR primers	GTAACCCGTTGAACCCCATT	CCATCCAATCGGTAGTAGCG
V4 region of the 16S rRNA gene	GTGYCAGCMGCCGCGGTAA (515F, Parada)	GGACTACNVGGGTWTCTAAT (806R, Apprill)
Software		
ImageJ	National Institute of Health	https://imagej.net/Welcome
Prism 9	GraphPad	https://www.graphpad.com/scientific-software/prism/
Molsoft	Molsoft, LLC	https://www.molsoft.com/index.html
LAS-X	Leica	www.leica-microsystems.com/products/microscope-software/p/leica-las-x-ls
SnapGene	GSL Biotech LLC	https://www.snapgene.com/
Illustrator	Adobe	https://www.adobe.com/products/illustrator.html
ImageStudio Lite	LI-COR	https://www.licor.com/bio/image-studio-lite/
ClueGO	Cytoscape	https://academic.oup.com/bioinformatics/article/25/8/1091/324247
NetworkX	Python	https://networkx.org
Gephi	Gephi	https://gephi.org
Plasmid		
pET-28b-GIV-CT-WT (aa 1660-1870)	(37)	Referred to as His-GIV-CT (aa 1660-1870)
pGEX-4T-GIV-CT-WT (a.a. 1623-1870)	(37)	Referred to as GST-GIV-CT (aa 1660-1870)

pCEFL-GST-GIV-CT (1660-1870)	(38)	Referred to as pCEFL GST-GIV-CT for mammalian expression (aa 1660-1870)
CMV14-p3X FLAG-GIV WT (full length)	(37)	N/A
pcDNA5-FRT-TO-RIP2-WT	(39)	Addgene 131201
HA-tagged NOD2-WT	From Dana J. Philpott (University of Toronto, Canada); originally from M. D'Amato (Karolinska Institute) (24)	NA
HA-tagged NOD1	From Dana J. Philpott (University of Toronto, Canada); originally from M. D'Amato (Karolinska Institute) (24)	NA
His-Myc-tagged NOD WT	From Santanu Bose (Washington State University, USA) (26)	NA
His-Myc-tagged NOD2 Δ CARD	From Santanu Bose (Washington State University, USA) (26)	NA
His-Myc-tagged NOD2 Δ NBD	From Santanu Bose (Washington State University, USA) (26)	NA
His-Myc-tagged NOD2 Δ LRR	From Santanu Bose (Washington State University, USA) (26)	NA
HA-tagged NOD2-1007fs	*This paper*	aa numbering according to Protein hNOD2 : UniProtKB/Swiss-Prot: Q9HC29.1
HA-tagged NOD2-G908R	*This paper*	aa numbering according to Protein hNOD2 : UniProtKB/Swiss-Prot: Q9HC29.1
HA-tagged NOD2-R702W	*This paper*	aa numbering according to Protein hNOD2 : UniProtKB/Swiss-Prot: Q9HC29.1
HA-tagged NOD2-R1034A	*This paper*	aa numbering according to Protein hNOD2 : UniProtKB/Swiss-Prot: Q9HC29.1
HA-tagged NOD2-R1037A	*This paper*	aa numbering according to Protein hNOD2 : UniProtKB/Swiss-Prot: Q9HC29.1
HA-tagged NOD2-LRR- delΔ (aa 1006-1040)	*This paper*	aa numbering according to Protein hNOD2 : UniProtKB/Swiss-Prot: Q9HC29.1
pCMV-Myc-tagged NOD2-FL-WT (Cloning site: XhoI/NotI)	*This paper*	aa numbering according to Protein hNOD2 : UniProtKB/Swiss-Prot: Q9HC29.1
pGEX-6P-2-NOD2-LRR-WT (aa 744-1040) (Cloning site: BamHI/XhoI)	*This paper*	aa numbering according to Protein hNOD2 : UniProtKB/Swiss-Prot: Q9HC29.1
pGEX-6P-2-NOD2-LRR-R1034A LRR (aa 744-1040)	*This paper*	aa numbering according to Protein hNOD2 : UniProtKB/Swiss-Prot: Q9HC29.1
pGEX-6P-2-NOD2-LRR-R1037A LRR (aa 744-1040)	*This paper*	aa numbering according to Protein hNOD2 : UniProtKB/Swiss-Prot: Q9HC29.1
pGEX-6P-2-NOD2-LRR- delΔ (aa 1006-1040)	*This paper*	aa numbering according to Protein hNOD2 : UniProtKB/Swiss-Prot: Q9HC29.1

Supplementary References

1. Sahoo D, et al. Extracting binary signals from microarray time-course data. *Nucleic Acids Res.* 2007;35(11):3705-12.
2. Sahoo D, et al. Artificial intelligence guided discovery of a barrier-protective therapy in inflammatory bowel disease. *Nat Commun.* 2021;12(1):4246.
3. Kanehisa M, and Goto S. KEGG: kyoto encyclopedia of genes and genomes. *Nucleic Acids Res.* 2000;28(1):27-30.
4. Avila Cobos F, et al. Benchmarking of cell type deconvolution pipelines for transcriptomics data. *Nat Commun.* 2020;11(1):5650.
5. Monaco G, et al. RNA-Seq Signatures Normalized by mRNA Abundance Allow Absolute Deconvolution of Human Immune Cell Types. *Cell Rep.* 2019;26(6):1627-40.e7.
6. Pavlou S, et al. Higher phagocytic activity of thioglycollate-elicited peritoneal macrophages is related to metabolic status of the cells. *J Inflamm (Lond).* 2017;14:4.
7. Das S, et al. Brain angiogenesis inhibitor 1 (BAI1) is a pattern recognition receptor that mediates macrophage binding and engulfment of Gram-negative bacteria. *Proc Natl Acad Sci U S A.* 2011;108(5):2136-41.
8. Sarkar A, et al. ELMO1 Regulates Autophagy Induction and Bacterial Clearance During Enteric Infection. *J Infect Dis.* 2017;216(12):1655-66.
9. Sayed IM, et al. Host engulfment pathway controls inflammation in inflammatory bowel disease. *Febs j.* 2020;287(18):3967-88.
10. Asai M, et al. Similar phenotypes of Girdin germ-line and conditional knockout mice indicate a crucial role for Girdin in the nestin lineage. *Biochem Biophys Res Commun.* 2012;426(4):533-8.
11. Swanson L, et al. TLR4 signaling and macrophage inflammatory responses are dampened by GIV/Girdin. *Proc Natl Acad Sci U S A.* 2020;117(43):26895-906.
12. Thompson LR, et al. A communal catalogue reveals Earth's multiscale microbial diversity. *Nature.* 2017;551(7681):457-63.
13. de Muinck EJ, et al. A novel ultra high-throughput 16S rRNA gene amplicon sequencing library preparation method for the Illumina HiSeq platform. *Microbiome.* 2017;5(1):68.
14. Walters W, et al. Improved Bacterial 16S rRNA Gene (V4 and V4-5) and Fungal Internal Transcribed Spacer Marker Gene Primers for Microbial Community Surveys. *mSystems.* 2016;1(1).
15. Marotz C, et al. Triplicate PCR reactions for 16S rRNA gene amplicon sequencing are unnecessary. *Biotechniques.* 2019;67(1):29-32.
16. Bolyen E, et al. Reproducible, interactive, scalable and extensible microbiome data science using QIIME 2. *Nat Biotechnol.* 2019;37(8):852-7.
17. Callahan BJ, et al. DADA2: High-resolution sample inference from Illumina amplicon data. *Nat Methods.* 2016;13(7):581-3.
18. Lahti L, and Shetty S. Tools for microbiome analysis in R. Version http://microbiome.github.com/microbiome.
19. Bhinder G, et al. The Citrobacter rodentium mouse model: studying pathogen and host contributions to infectious colitis. *J Vis Exp.* 2013(72):e50222.
20. Koroleva EP, et al. Citrobacter rodentium-induced colitis: A robust model to study mucosal immune responses in the gut. *J Immunol Methods.* 2015;421:61-72.
21. Chassaing B, et al. Dextran sulfate sodium (DSS)-induced colitis in mice. *Curr Protoc Immunol.* 2014;104:Unit 15 25.
22. Kiesler P, et al. Experimental Models of Inflammatory Bowel Diseases. *Cell Mol Gastroenterol Hepatol.* 2015;1(2):154-70.
23. Kim JJ, et al. Investigating intestinal inflammation in DSS-induced model of IBD. *J Vis Exp.* 2012(60).
24. Travassos LH, et al. Nod1 and Nod2 direct autophagy by recruiting ATG16L1 to the plasma membrane at the site of bacterial entry. *Nat Immunol.* 2010;11(1):55-62.
25. Linderson Y, et al. Functional interaction of CARD15/NOD2 and Crohn's disease-associated TNFalpha polymorphisms. *Int J Colorectal Dis.* 2005;20(4):305-11.
26. Sabbah A, et al. Activation of innate immune antiviral responses by Nod2. *Nat Immunol.* 2009;10(10):1073-80.
27. Ghosh P, et al. Activation of Galphai3 triggers cell migration via regulation of GIV. *J Cell Biol.* 2008;182(2):381-93.

28. Ghosh P, et al. A Galphai-GIV molecular complex binds epidermal growth factor receptor and determines whether cells migrate or proliferate. *Mol Biol Cell.* 2010;21(13):2338-54.
29. Dyer BW, et al. A noncommercial dual luciferase enzyme assay system for reporter gene analysis. *Anal Biochem.* 2000;282(1):158-61.
30. Newman JV, et al. Citrobacter rodentium espB is necessary for signal transduction and for infection of laboratory mice. *Infect Immun.* 1999;67(11):6019-25.
31. Lu Y, et al. Palmitoylation of NOD1 and NOD2 is required for bacterial sensing. *Science.* 2019;366(6464):460-7.
32. Lécine P, et al. The NOD2-RICK complex signals from the plasma membrane. *J Biol Chem.* 2007;282(20):15197-207.
33. Vijayrajratnam S, et al. Understanding the molecular differential recognition of muramyl peptide ligands by LRR domains of human NOD receptors. *Biochem J.* 2017;474(16):2691-711.
34. Darfeuille-Michaud A, et al. High prevalence of adherent-invasive Escherichia coli associated with ileal mucosa in Crohn's disease. *Gastroenterology.* 2004;127(2):412-21.
35. Schauer DB, and Falkow S. Attaching and effacing locus of a Citrobacter freundii biotype that causes transmissible murine colonic hyperplasia. *Infect Immun.* 1993;61(6):2486-92.
36. Holgersen EM, et al. Transcriptome-Wide Off-Target Effects of Steric-Blocking Oligonucleotides. *Nucleic Acid Ther.* 2021;31(6):392-403.
37. Garcia-Marcos M, et al. GIV is a nonreceptor GEF for G alpha i with a unique motif that regulates Akt signaling. *Proc Natl Acad Sci U S A.* 2009;106(9):3178-83.
38. Bhandari D, et al. Cyclin-dependent kinase 5 activates guanine nucleotide exchange factor GIV/Girdin to orchestrate migration-proliferation dichotomy. *Proc Natl Acad Sci U S A.* 2015;112(35):E4874-83.
39. Ellwanger K, et al. XIAP controls RIPK2 signaling by preventing its deposition in speck-like structures. *Life Sci Alliance.* 2019;2(4).
