# Supplementary material for: Distinct colitis-associated macrophages drive NOD2-dependent bacterial sensing and gut homeostasis
Source: J Clin Invest. 2025 Oct 2;135(23):e190851. doi: 10.1172/JCI190851 (PMC12646664; doi:10.1172/JCI190851)

Full unedited blot for Figure 2A

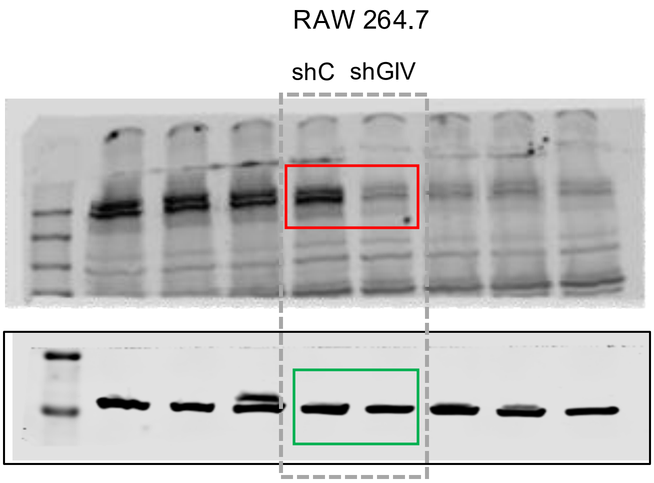

Full unedited blot for Figure 2D

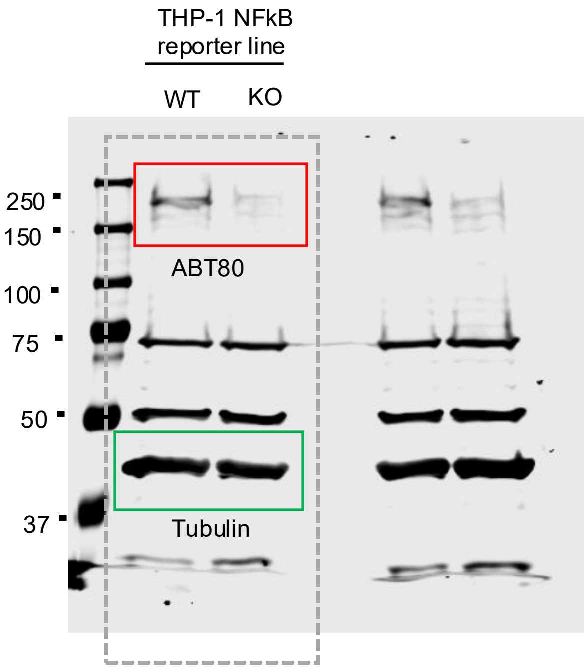

Full unedited blot for Figure 2J

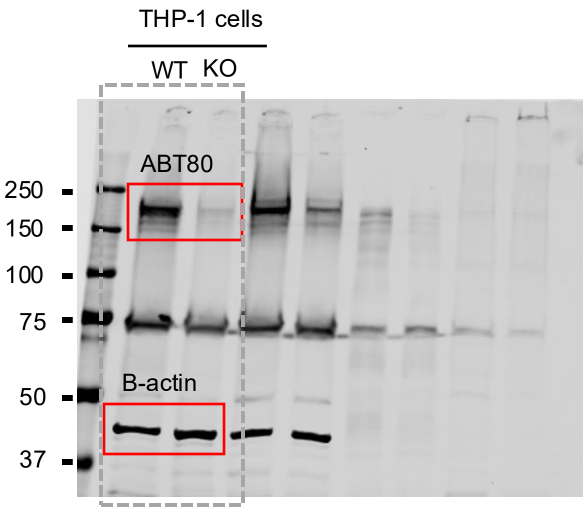

Full unedited blot for Figure 7B

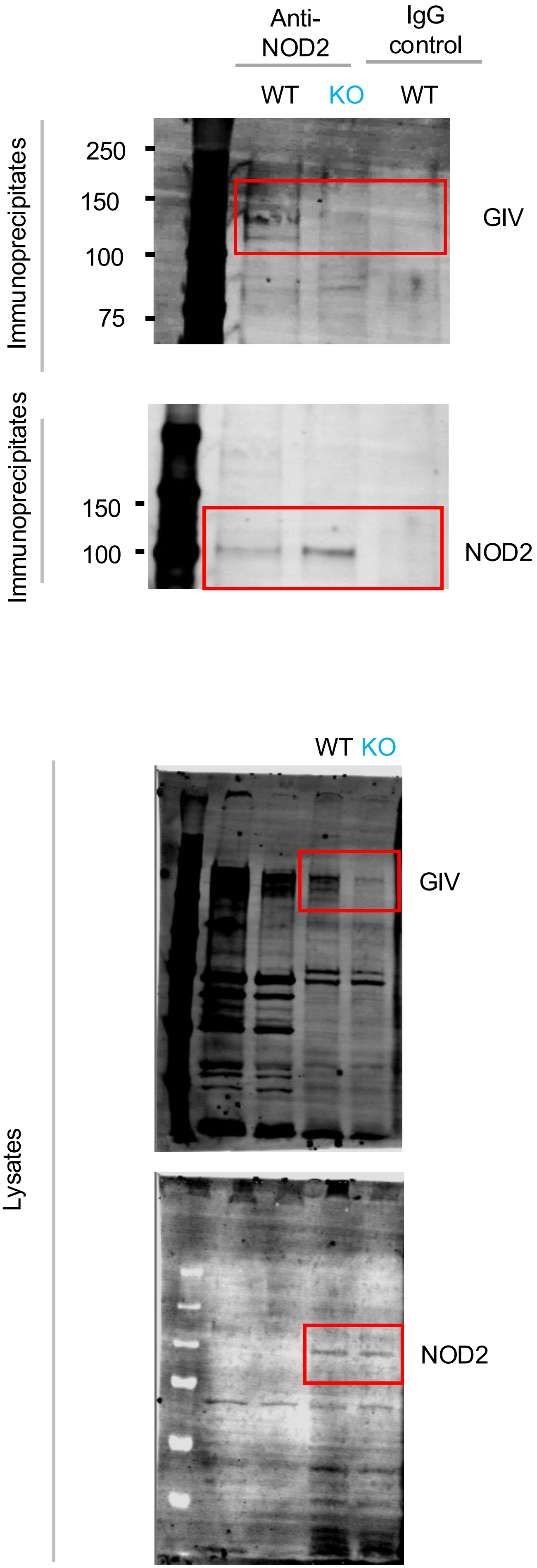

Full unedited blot for Figure 7G

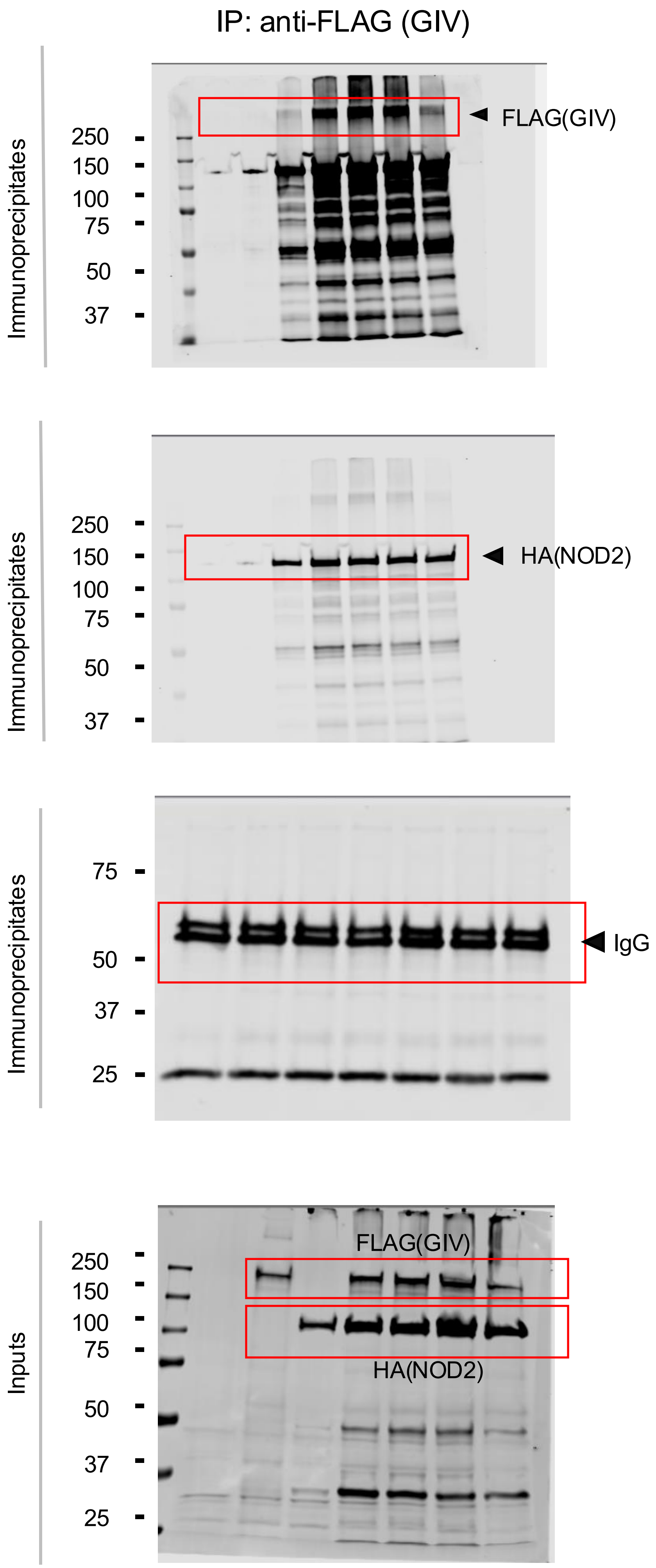

Full unedited blot for Figure 8C

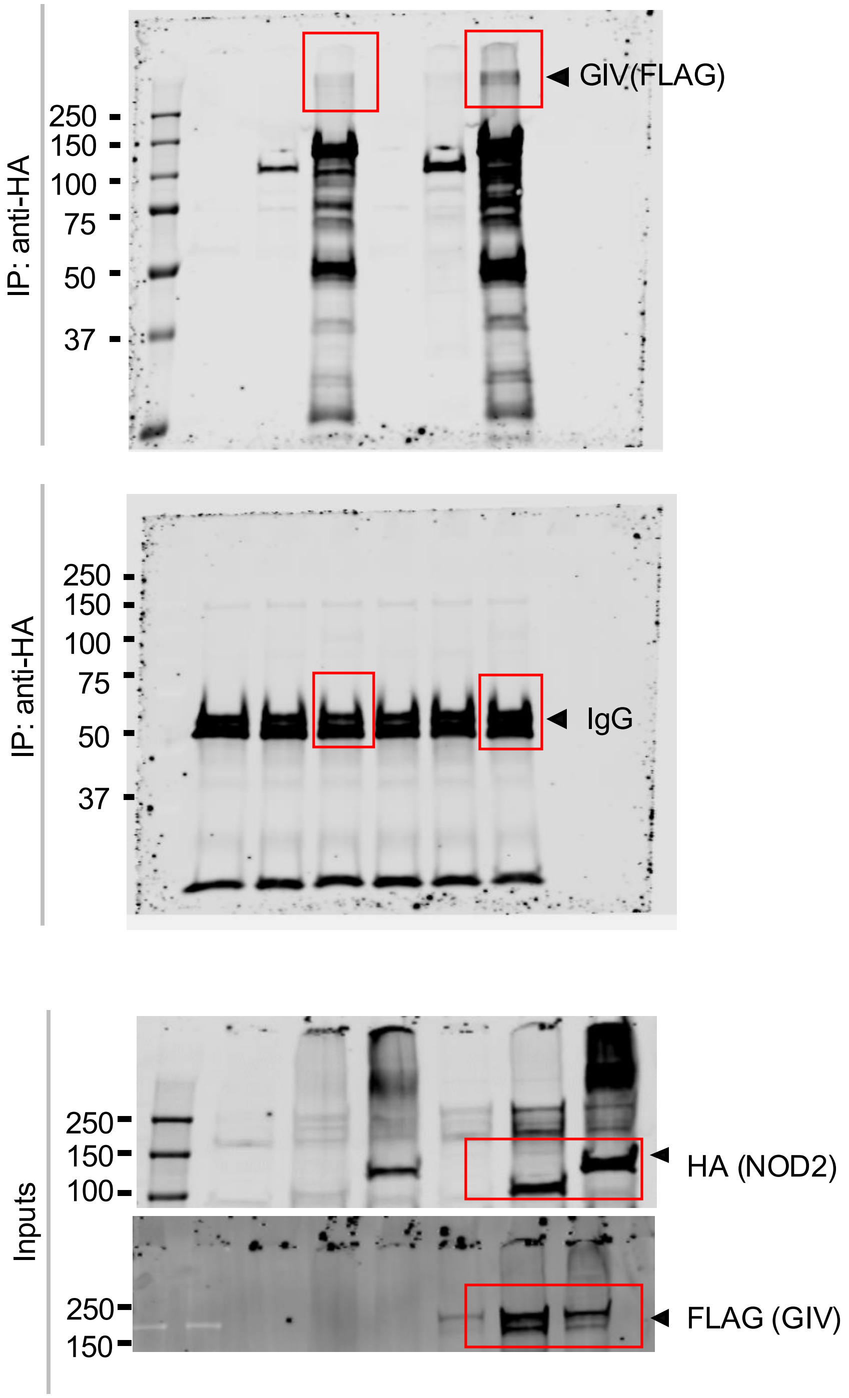

Full unedited blot for Figure 8G

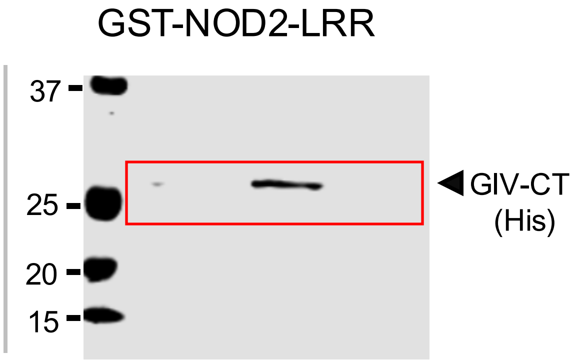

Full unedited blot for Figure 8F

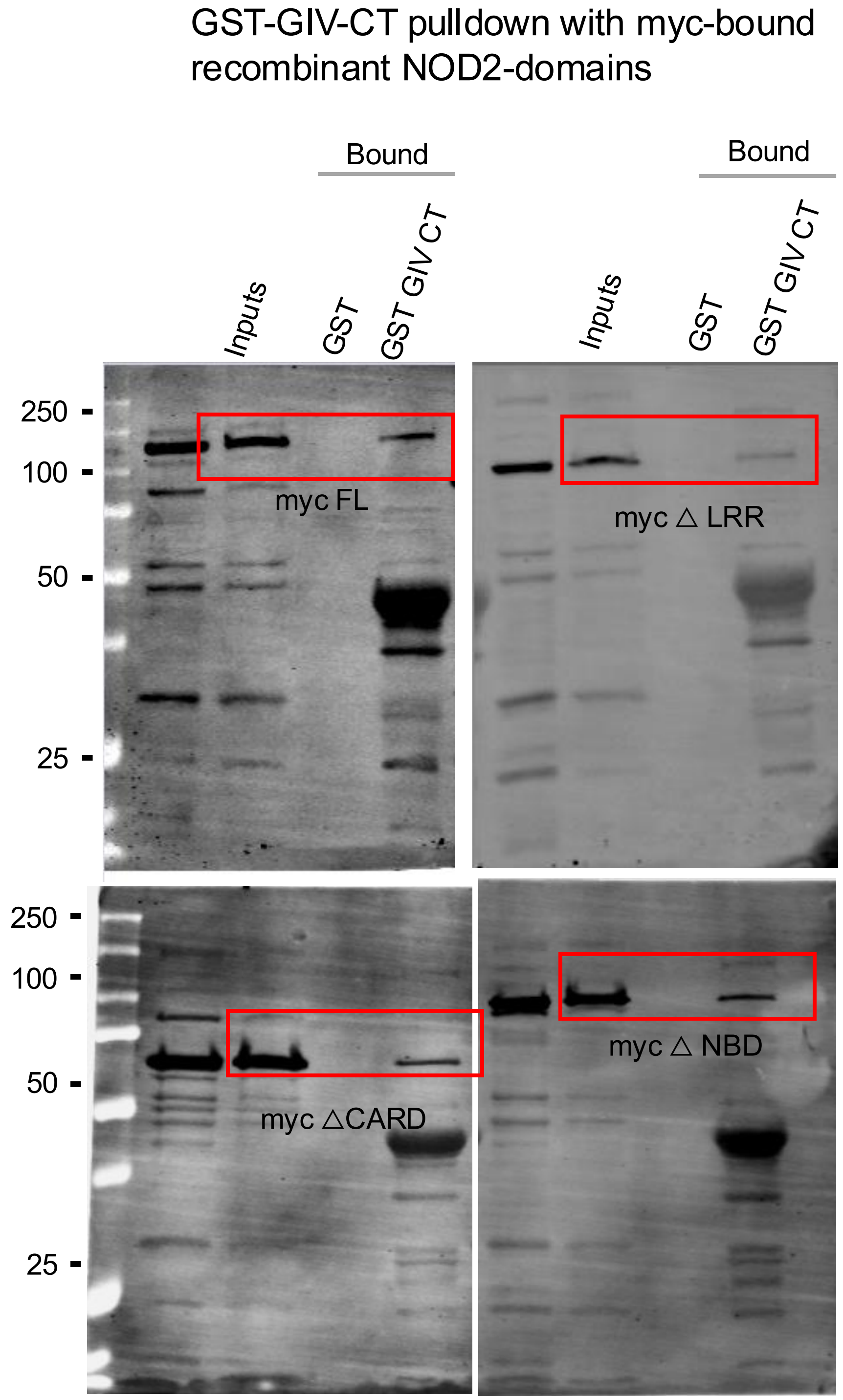

Full unedited blot for Figure 8I

GST-GIV-CT pulldown with lysates of HEK293T

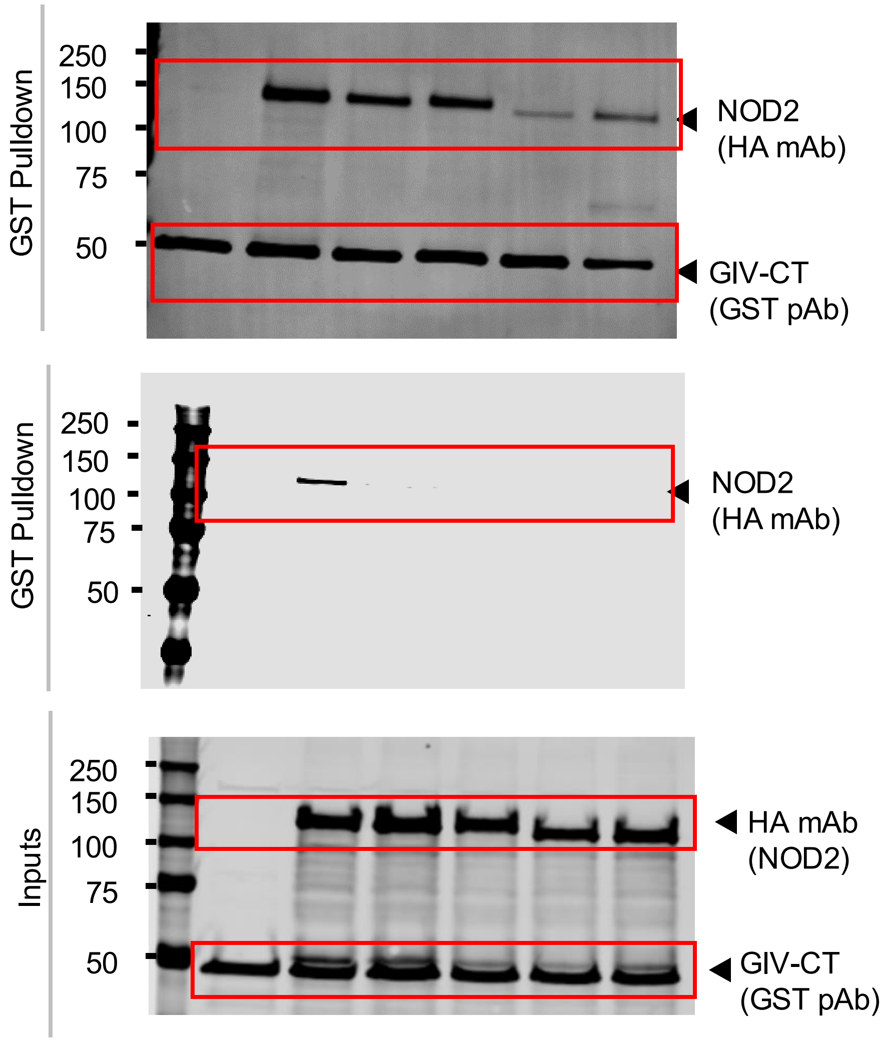

Full unedited blot for Figure 9C

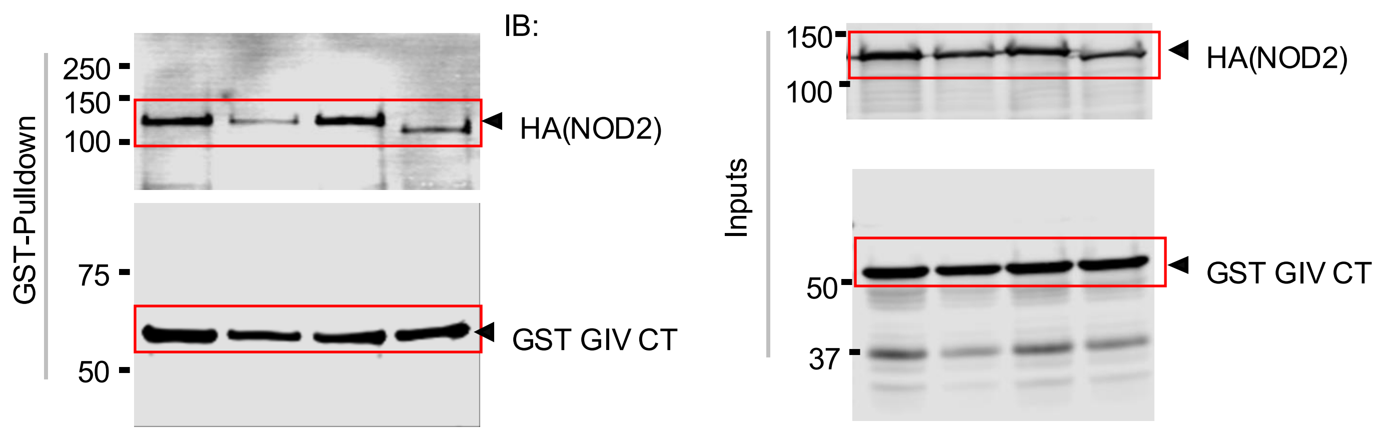

Full unedited blot for Figure 9D

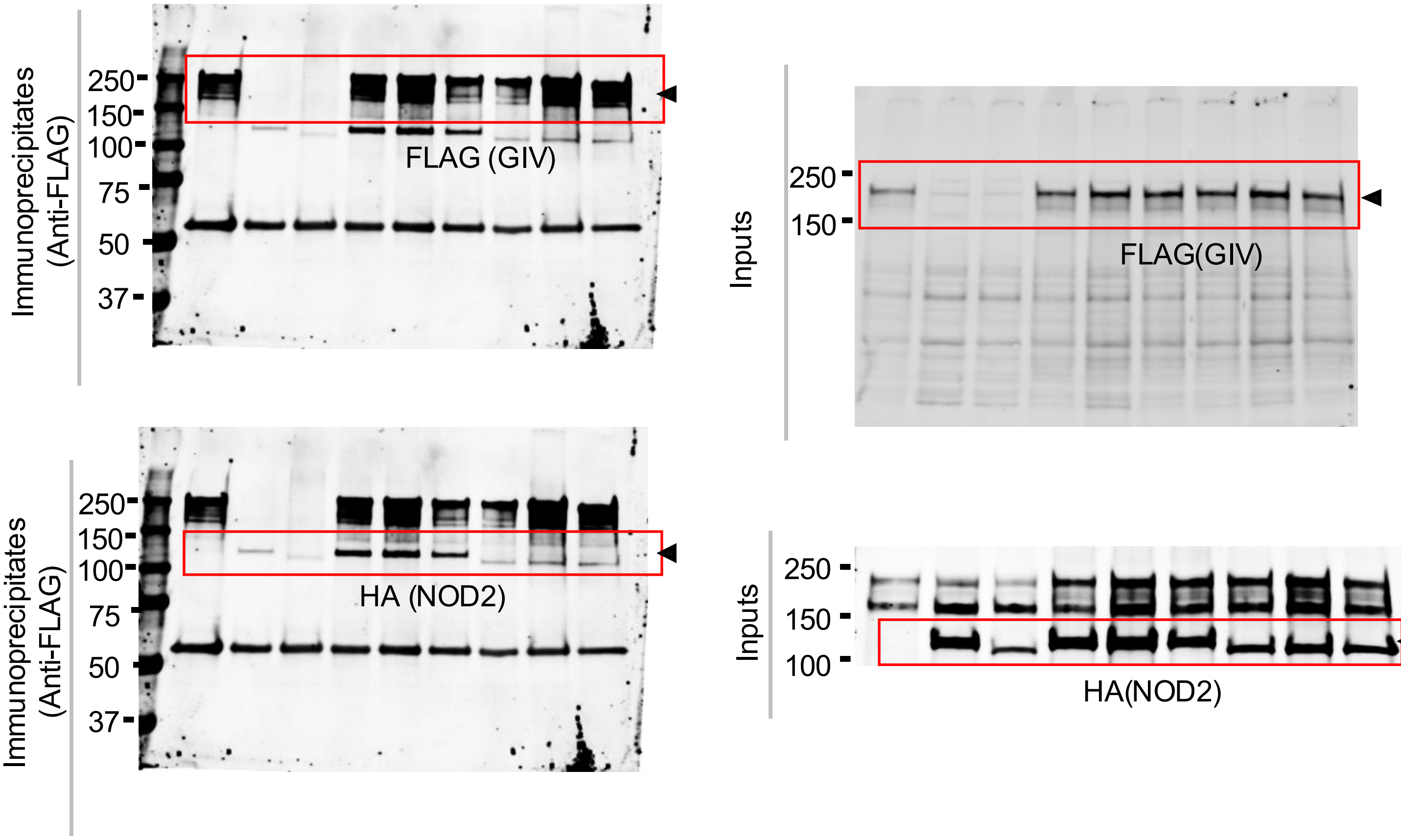

Full unedited blot for Supplementary Figure 1B

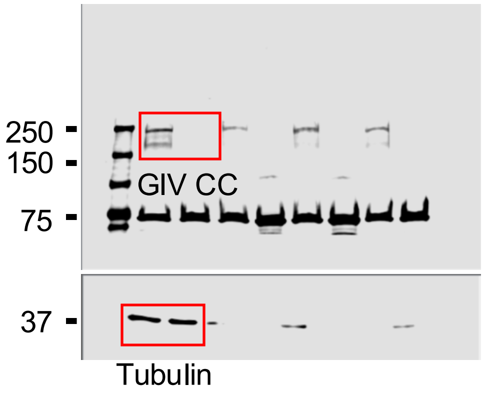

Full unedited blot for Supplementary Figure 4A

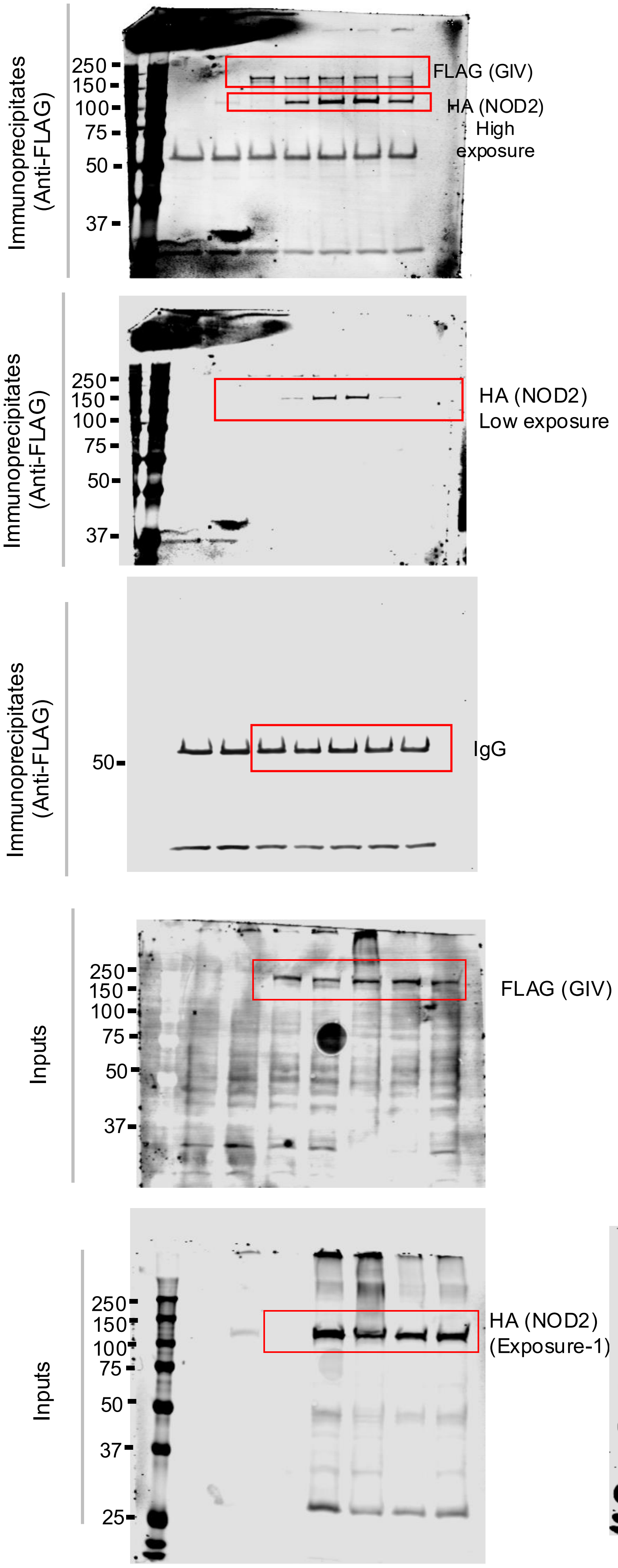

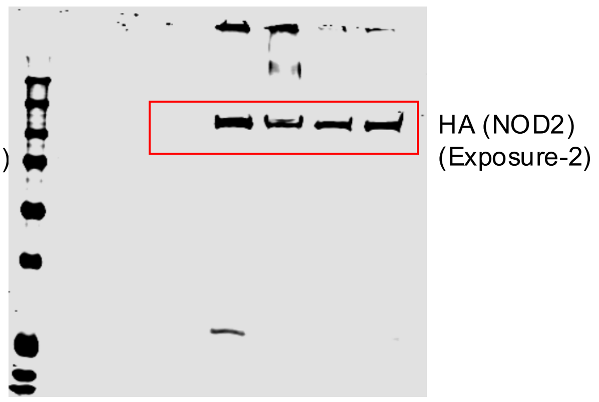

Full unedited blot for Supplementary figure 4B

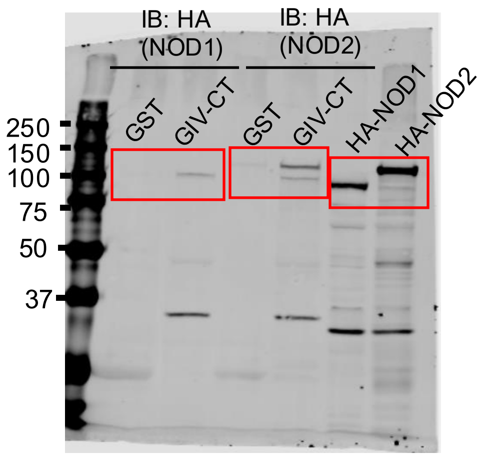

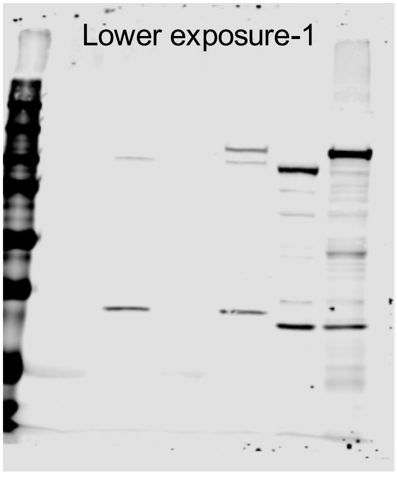

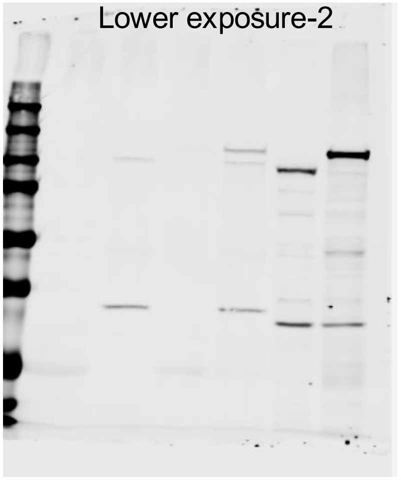

Full unedited blot for Supplementary figure 4C

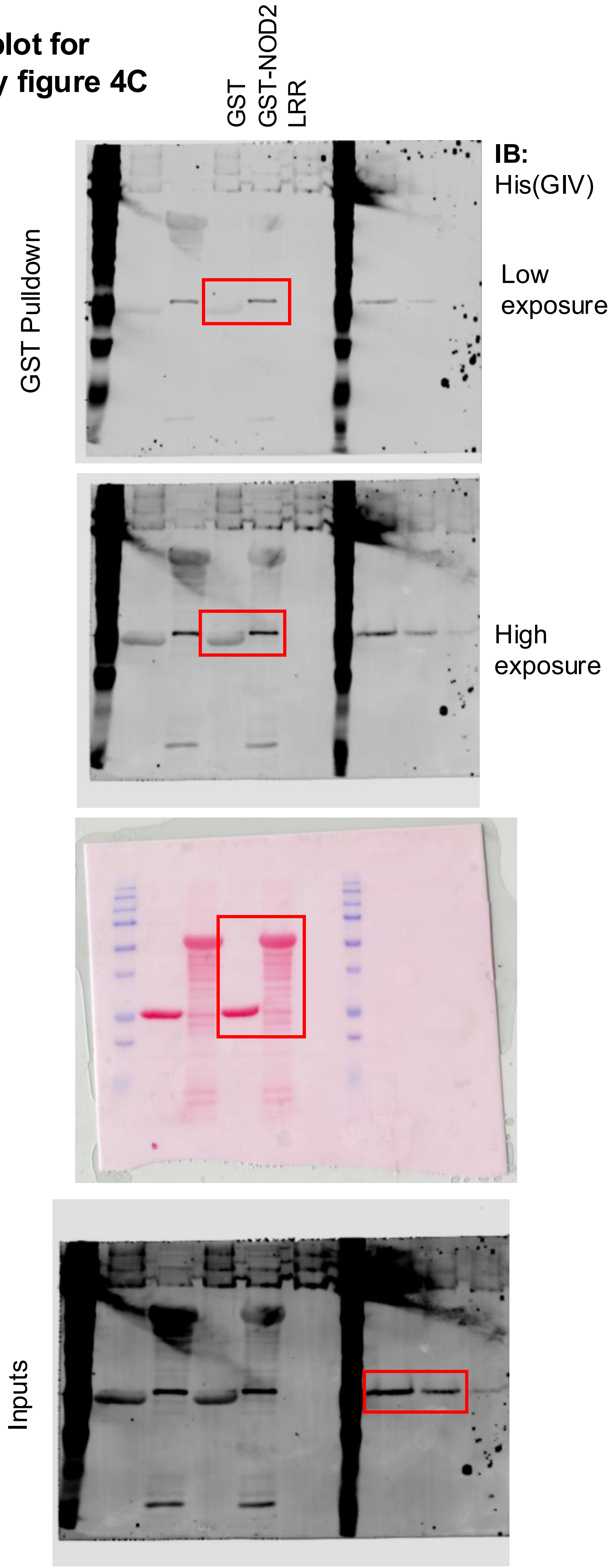

Supplementary figure 4D

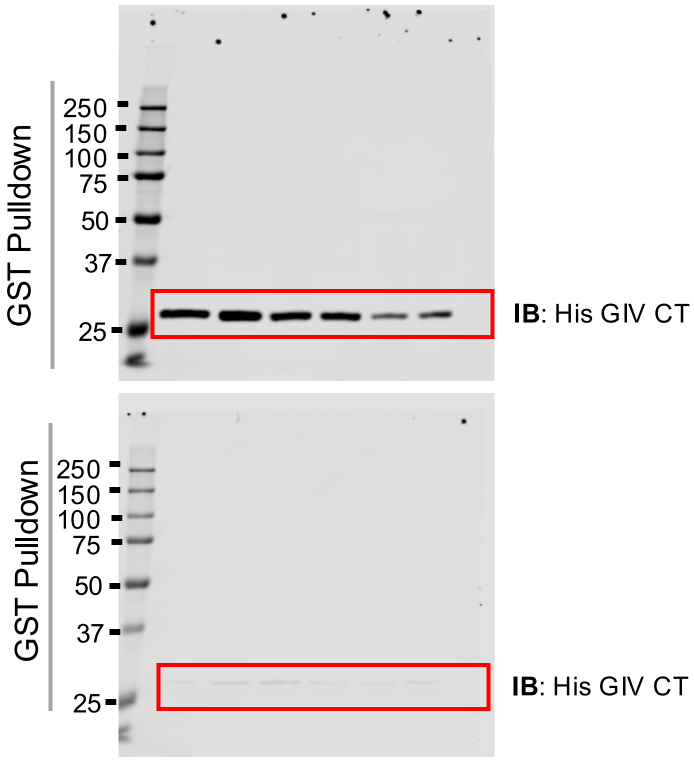

Full unedited blot for Supplementary figure 4E

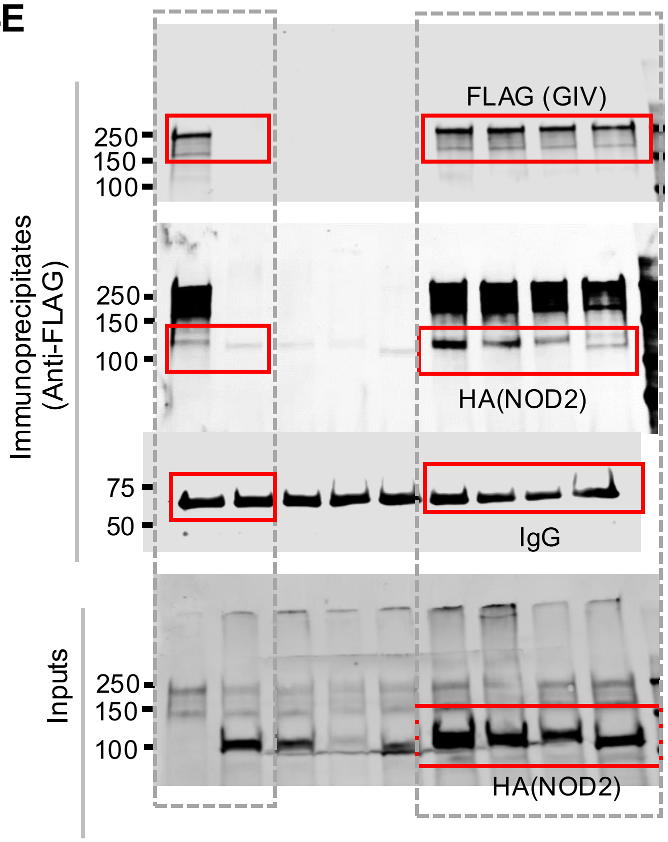

Supplementary figure 4F

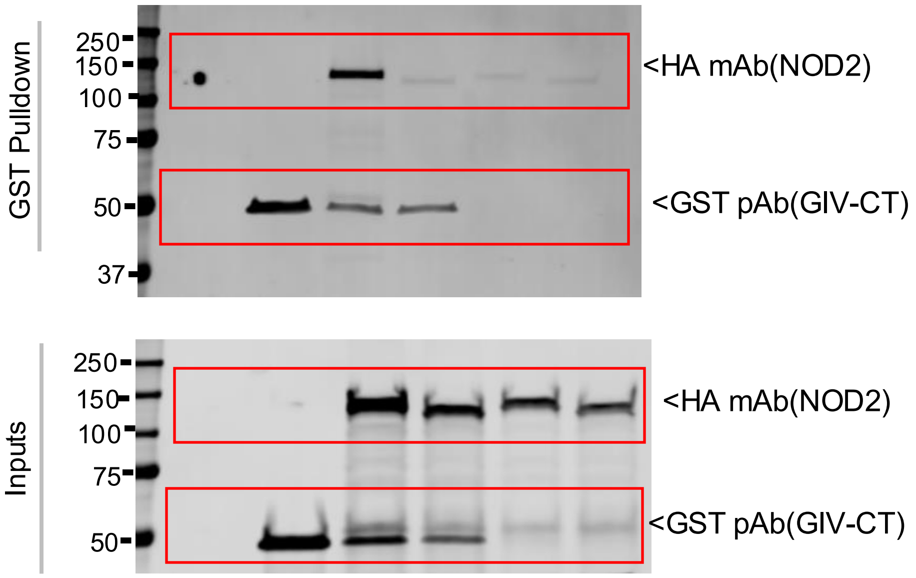

Supplement: Unedited blot and gel images [file jci-135-190851-s191.pdf]
